# Differentiation and localization of interneurons in the developing spinal cord depends on DOT1L expression

**DOI:** 10.1186/s13041-020-00623-3

**Published:** 2020-05-29

**Authors:** Angelica Gray de Cristoforis, Francesco Ferrari, Frédéric Clotman, Tanja Vogel

**Affiliations:** 1 Department of Molecular Embryology, Institute of Anatomy and Cell Biology, Faculty of Medicine, Albert-Ludwigs-University Freiburg, 79104 Freiburg, Germany; 2 Spemann Graduate School of Biology and Medicine (SGBM), Albert-Ludwigs-University Freiburg, 79104 Freiburg, Germany; 3 Faculty of Biology, Albert-Ludwigs-University Freiburg, 79104 Freiburg, Germany; 4 Max Planck Institute of Immunobiology and Epigenetics, 79108 Freiburg, Germany; 5 Laboratory of Neural Differentiation, Institute of Neuroscience, Université catholique de Louvain, Brussels, Belgium; 6 Centre for Basics in Neuromodulation (Neuromodul Basics), Freiburg, Germany

**Keywords:** Spinal cord, Interneuron, Specification, Localization, Methyltransferase, DOT1L, Epigenetics

## Abstract

Genetic and epigenetic factors contribute to the development of the spinal cord. Failure in correct exertion of the developmental programs, including neurulation, neural tube closure and neurogenesis of the diverse spinal cord neuronal subtypes results in defects of variable severity. We here report on the histone methyltransferase Disruptor of Telomeric 1 Like (DOT1L), which mediates histone H3 lysine 79 (H3K79) methylation. Conditional inactivation of DOT1L using Wnt1-cre as driver (*Dot1l*-cKO) showed that DOT1L expression is essential for spinal cord neurogenesis and localization of diverse neuronal subtypes, similar to its function in the development of the cerebral cortex and cerebellum. Transcriptome analysis revealed that DOT1L deficiency favored differentiation over progenitor proliferation. *Dot1l*-cKO mainly decreased the numbers of dI1 interneurons expressing *Lhx2*. In contrast, *Lhx9* expressing dI1 interneurons did not change in numbers but localized differently upon *Dot1l-*cKO. Similarly, loss of DOT1L affected localization but not generation of dI2, dI3, dI5, V0 and V1 interneurons. The resulting derailed interneuron patterns might be responsible for increased cell death, occurrence of which was restricted to the late developmental stage E18.5. Together our data indicate that DOT1L is essential for subtype-specific neurogenesis, migration and localization of dorsal and ventral interneurons in the developing spinal cord, in part by regulating transcriptional activation of *Lhx2*.

## Introduction

The central nervous system (CNS), composed of spinal cord and brain, forms through invagination of the neural ectoderm and fusion of the neural folds to generate the neural tube, in a process called neurulation. Fusion of the neural folds and closure of the neural tube is completed between E8.75 and E10 in the mouse [1]. A delicate molecular network tightly orchestrates the development of the spinal cord in its entire complexity of different cell types. Many factors take part in the precise processes that initiate and maintain cell-specific transcriptional profiles, which are necessary for both neurulation and the subsequent specification of the diverse neuronal subpopulations during neural tube development.

Opposite gradients of signaling pathways, for example those of Bone Morphogenetic Protein (BMP) and Wingless-related integration site (WNT) from the roofplate, or Sonic Hedgehog (SHH) from the floorplate, tightly regulate the establishment of diverse progenitor classes along the dorsoventral axis [2]. According to different marker gene expression patterns and response to signaling molecules, the developing spinal cord is subdivided into six dorsal progenitor classes (dp1–6 interneuron progenitors) and five ventral progenitor classes (p0–3 interneuron progenitors and pMN motor neurons progenitors). Two waves of neurogenesis lead to the differentiation of the progenitor classes into mature interneurons or motor neurons (MN) [3]. In the first wave, which takes place between E9.0-E12.5, dorsal interneuron classes (dI1–6) [4–9] and ventral interneurons (V0–3) differentiate along with MN [10–15], each from the respective progenitor class. A smaller subset of dorsal interneurons, dIL_A_ and dIL_B,_ is generated in the second wave between E11.5 and E13.5 [6, 9, 16, 17]. Maturing interneurons migrate from the ventricular zone into the mantle zone to their final position to build and to integrate into functional neuronal circuits. The dorsal populations dI1-dI4 and dIL_A_ integrate into circuits involved in proprioception or touch-related motor control [4, 18, 19], dI4/dIL_A_ and dI5/dIL_B_ populations into networks transmitting information for pain, thermal sensation, itch and touch [20–23]. Mature dI6 interneurons control alternating hindlimb movement and coordination [24, 25]. The ventrally generated interneuron classes integrate into locomotor networks, largely projecting on MNs [3]: different subsets of V0 participate in locomotion [26] and coordination of left-right alternation [27], V1 and V2 contribute to limb articulation [28], and V3 to modulation of the locomotion rhythm [29]. The MNs, arising from a single progenitor class, differentiate into a wide range of functionally diversified mature classes. MNs located in the spinal cord and brainstem are cholinergic and target a variety of muscles [30].

Both impaired neurulation and neurogenesis during spinal cord development lead to diverse pathologies. Defects in neural tube fusion result in NTDs, which are among the most common birth defects in human pregnancies. The range of severity spans from lethal (anencephaly, craniorachischisis) to neurological handicaps (open spina bifida) to asymptomatic conditions (closed spina bifida) [31]. Further, defective neurogenesis and patterning can lead to neuropathies [2] due to malformed circuitry regarding touch [20], itch [32] and pain [33].

Both genetic and epigenetic factors have been studied to identify the etiology of NTDs, especially since folate deficiency has been indicated as a cofactor of NTD occurrence [34]. Recent studies suggested that histone modifications such as acetylation or methylation correlated with the occurrence of NTD in mouse or chick embryos [35–39]. Further, chromatin modifications have been linked to defective neurogenesis and patterning of the embryonic spinal cord [40–42]. But although epigenetic modifiers have the ability to confer differential transcriptional programs that direct stem cells along specific developmental trajectories, for many chromatin-modifying enzymes, especially impacting on histone methylation, functions with regard to embryonic spinal cord development are still unresolved. Histone H3 lysine methylation occurs at different positions including K4, K9 or K27. Recently, modifications of K79 were linked with NTD. Specifically, attenuation of H3K79 dimethylation (H3K79me2) correlated with NTD occurrence in brain samples of human embryos presenting with spina bifida [43]. Further, high levels of H3K79 homocysteinylation (Hcy), in place of methylation at this position, correlated with decreased expression of specific genes, e.g. S*marca4*, *Cecr2* and *Dnmt3b*, which are known to cause NTD upon disbalanced transcription [44]. H3K79 mono-, di- and trimethylation (H3K79 me1, me2 or me3) is mainly conferred by the Disruptor of Telomeric Silencing 1 Like (DOT1L) in mammals [45]. DOT1L function has been linked to a range of cellular processes including transcriptional activation, resume of transcription after DNA damage, or cell cycle progression [45]. DOT1L participates in different protein complexes, which might explain its diverse functionalities [46] and it is involved in specific forms of leukemia [47, 48]. It is also indispensable for development [49], as loss of DOT1L is lethal at very early embryonic stages in the mouse [50]. DOT1L is fundamental for development of diverse organs, as shown for example for cardiomyocytes differentiation [51] and functionality [52], erythropoiesis [53], and chondrogenesis [54]. Previous studies from our laboratory highlighted that DOT1L activity plays important functional roles for CNS development. In the cerebral cortex and cerebellum, but also in other somatic organ systems, DOT1L balances progenitor proliferation and differentiation [55–58].

We here report on our study aiming to explore the role of DOT1L in neural tube development. Using Wnt1-cre mediated conditional deletion of DOT1L in the developing mouse neural tube, we here show that in this model system DOT1L is not affecting neural tube closure, but that it is involved in correct differentiation of specific subsets of interneurons. Specifically, DOT1L contributes to the molecular specification of LHX2-expressing dI1 interneurons and migration of the LHX9-expressing dI1 subset together with dI2, dI3, V0 and V1.

## Materials and methods

### Mice

Wnt1-cre animals (Wnt1cre2 [59]) were mated with floxed *Dot1l*. Animals with the genotype Wnt1^cre/+^;Dot1l^flox/flox^ (cKO) were analyzed in comparison to littermates with Wnt1^+/+^;Dot1l^flox/+^, Wnt1^+/+^;Dot1l^flox/flox^ or Wnt1^cre/+^;Dot1l^flox/+^ as controls. For EdU pulse labeling of progenitors, pregnant females were injected at E11.5 with 140 μg/g body weight EdU (C10337, Invitrogen, PA, USA) and embryos were harvested 30 min later. Animal experiments were approved by animal welfare committees of the University of Freiburg and local authorities (G16/069).

### In situ hybridization (ISH), immunostainings and histological stainings (Nissl, Hematoxylin-eosin)

ISH was performed following published protocols [60]. The probes used in the present study are reported in the Supplementary methods (Table S1). Immuno- and histological staining procedures, used antibodies (Table S2), imaging and quantification of stainings are reported in the Supplementary methods.

### RNA-seq

Mouse embryos at E12.5 were dissected in ice-cold DPBS (14190–094, Gibco, MA, USA). Lumbar spinal cords were isolated and flash-frozen in liquid nitrogen. 5 controls and 5 mutant littermates, coming from two litters, were thawed and RNA was extracted using RNAeasy Mini kit (#74106, Qiagen, Hilden, Germany), and separate libraries were generated using the NEBNext Ultra RNA Library Prep Kit for Illumina following manufacturer’s instructions (#E7530S/L, NEB, Frankfurt, Germany). The libraries were sequenced with 40 Mio reads per sample, paired end and read length of 75 bp.

### Bioinformatics analysis of RNA-seq

Raw data from the Illumina HiSeq 4000 sequencing machine (running a HiSeq Control Software HD 3.4.0.38) was demultiplexed and converted into FASTQ files using Illumina bcl2fastq2.17 v2.17.1.14. Quality control, mapping and gene-level quantification were generated using the RNA-seq workflow of snakepipes (v. 1.2.2) [61], using default parameters. Briefly, read quality was assessed using FastQC (v. 0.11.8) (https://www.bioinformatics.babraham.ac.uk/projects/fastqc/), trimmed using TrimGalore (v. 0.5.0) (https://www.bioinformatics.babraham.ac.uk/projects/trim_galore/) and mapped to the genome build mm10 using STAR (v. 2.6.1d) [62]. Gene-level quantification was obtained using featureCounts from the Subread suite (v. 1.6.4) [63] on the gencode annotation M18. Downstream analysis was run with R (v. 3.5.2) and Python (v. 3.6). Differential expression analysis was performed on the count matrix using DESeq2 (v. 1.22.1) [64]. The general linear model was used to control for litter effects. To further visualize expression dynamics (either with scatter plots or heatmaps), we normalized our count matrix using transcripts per kilobase million (TPM), and we used comBat from the sva package (v. 3.30.1) [65] to correct for the litter effects. Visualizations were generated using the seaborne module (v. 0.8.1) in Python. Gene Ontology (GO) term enrichment analysis was performed using clusterProfiler (v. 3.10.1) [66].

### Analysis of cell distribution

Images of the different cell populations were acquired using the Apotome (Zeiss, Germany) setup and processed on Inkscape for counting and quantification by selecting first lumbar hemi-sections for analysis in Fiji-ImageJ. We measured the maximal height (from the ventral lower border of the spinal cord under the central canal to the dorsal-most border of the spinal cord) and width (from the center of the central canal to the most lateral edge) of each hemi-section for normalization [67]. Cells positive for the respective staining were marked and for each of them the distance and angle from the ventral-most point of the spinal cord (origin) were recorded. These measurements were plotted using Matlab into dorsoventral and mediolateral projections of the analyzed interneurons onto a hemi-section outline. The projections of each cell onto X and Y axes were exported from Matlab and the distributions were analyzed with a two-sample Hotelling’s T2 test as statistical tests using the NCSS12 software. Further information is reported in the extended Materials and Methods section (Figure S6).

## Results

### Wnt1-cre mediated DOT1L deficiency decreases transcription of dorsal progenitor marker genes during neurogenesis in the spinal cord

**Fig. 1:**
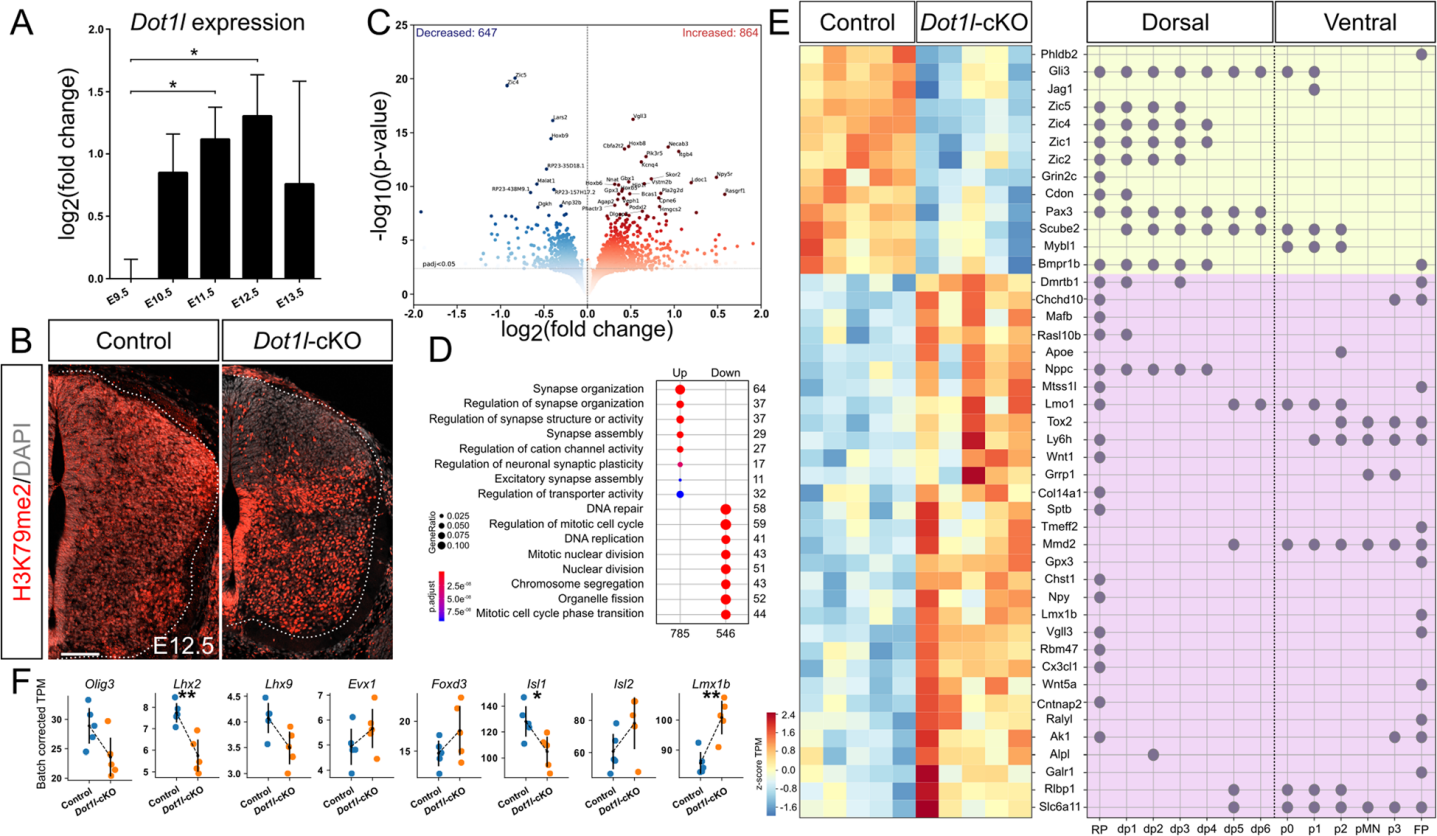
DOT1L activity during spinal cord neurogenesis supports progenitor maintenance and differentiation of dorsal interneurons. (**a**) qRTPCR analysis of *Dot1l* in wild type lumbar spinal cords at different embryonic stages (E9.5-E13.5) normalized to E9.5 (E9.5-E11.5 n = 3 from pooling of 3 individual embryos each, E12.5 and E13.5 n = 8 from individual embryos). qRTPCR represented with mean ± SEM. P-values were calculated with unpaired, two-tailed Student’s *t*-test: * *p* < 0.05 (**b**) Immunostaining H3K79me2 mark (red) and DAPI (gray) in E12.5 lumbar spinal cords in control and conditional *Dot1l* knock-out (*Dot1l*-cKO) embryos. Hemicord profile highlighted by dotted white line. Scale bar: 100 μm. (**c**) Volcano plot of differentially expressed genes (DEG) in *Dot1l*-cKO lumbar spinal cord compared to control littermates (*n* = 5) at E12.5. Colored, genes with increased (red) and decreased (blue) expression for adjusted *p* < 0.05. (**d**) GO term analysis for biological processes of DEG in *Dot1l*-cKO. Scales of gene ratio and adjusted p-value reported to the bottom left side. Genes with upregulated biological processes in left column, with downregulated biological processes upon *Dot1l*-cKO in right column. Number of genes per term on the Y axis of the graph, total numbers up- or down-regulated on X axis. Threshold for enrichment analysis: adjusted *p* < 0.1. (**e**, left panel) Heatmap for DEGs in mutant littermates intersected with a gene list for expressed progenitor domain-specific genes extrapolated from [68]. Color-coding based on TPM z-score, scale at the bottom top left side. (**e**, right panel) Annotation of progenitor domain-specificity relative to the genes intersected in the heatmap [68]. (**f**) Scatterplot for DEGs with batch corrected TPM for interneuron markers (*Olig3*, *Lhx2*, *Lhx9*, *Evx1*, *Foxd3*, *Isl1*, *Isl2*, *Lmx1b*) in control (blue) and mutant (orange) samples, each dot corresponding to a single n in the transcriptome analysis. Error bars represent SD in the transcriptome. Adjusted p-values from the DEG are reported as * adjusted *p* < 0.05, ** adjusted *p* < 0.01

A previous report proposed that H3K79me2 deposition impacts on spinal cord development and associates with pathologies including spina bifida [43]. To current knowledge, DOT1L is the main methyltransferase targeting H3K79 [45]. As the expression of *Dot1l* was previously not described over the course of mouse spinal cord development, we studied the expression of *Dot1l* throughout spinal cord neurogenesis, which occurs during E9.5 to E13.5. Using ISH, we observed transcription of *Dot1l* at all stages (Figure S1A). Signal intensity appeared stronger in the progenitor zone compared to the mantle zone. qRTPCR corroborated *Dot1l* expression at all stages examined. Compared to E9.5, which we set as baseline, *Dot1l* transcription increased significantly at E11.5 and E12.5 in the developing mouse spinal cord (Fig. 1a). Both time points are characterized by progenitor proliferation and generation of interneurons [6, 9]. This expression dynamic suggested that *Dot1l* might regulate neurogenesis during spinal cord development, as we observed also in other brain regions [55, 57]. Likewise, we studied *DOT1L* transcription in the developing chicken spinal cord. In chicken embryos, *DOT1L* expression was tested at three developmental stages (Hamilton Hamburger (HH) stages 11, 13+ and 16 comparable to murine E9.0, E9.5 and E10.0). Comparing *DOT1L* expression at HH13+ and HH16 to HH11, we observed increased transcript levels over time (Figure S1B).

To investigate the function of DOT1L during spinal cord development, we generated a transgenic mouse line by crossing floxed *Dot1l* with a Wnt1-Cre driver line, which is active in the developing spinal cord [59]. To assess the extent of *Cre* expression and its suggested activity towards inactivation of DOT1L in the conditional mouse mutant (*Dot1l-*cKO), we analyzed alteration of H3K79me2 patterns using immunostainings as a read-out for loss of DOT1L function. We focused our analyses on E12.5, where we observed highest levels of DOT1L expression, and on the lumbar spinal cord, where NTD like spina bifida are observable. In accordance with wide-spread transcription of *Dot1l* during spinal cord development (Figure S1A), H3K79me2 staining was uniformly observed in the entire lumbar area of control animals (Fig. 1b). In contrast, *Dot1l*-cKO littermates presented reduction of the H3K79me2 immunostaining mostly in dorsal areas, spanning the ventricular to the mantle zone (Fig. 1b). H3K79me2 was less homogenous in ventral regions in *Dot1l*-cKO than in control animals, suggesting either cell-specific cre-activity in ventral cells as well and/or dorsally derived *Dot1l*-cKO cells that intermingled with ventral cells not expressing cre. We visualized cre-activity by using a GFP-reporter allele (Figure S2A). Apparently, the GFP signal was detected in many cells along the dorsoventral and mediolateral axis of the spinal cord. The pattern suggested slightly stronger staining in dorsal regions compared to a scattered appearance of GFP-positive nuclei in the ventral domain. Stronger GFP staining in dorsal regions correlated with less intense H3K79me2 staining. We concluded that Wnt1-cre mediated DOT1L-deficiency might mostly affect dorsal but also ventral cell populations in lumbar areas of the spinal cord. We did not observe NTD in Wnt1-cre mediated *Dot1l*-cKO embryos at any time point during neurogenesis (Figure S1C), notwithstanding the indications that altered levels of H3K79me2 associate with closure defects of the neural tube in humans [43], and pharmacological inhibition of DOT1L increased occurrence of NTD in chicken (Figure S1D, E).

DOT1L-mediated histone methylation correlates with regulation of gene expression [52, 68]. In continuation of our initial expression analysis of *Dot1l-*cKO in lumbar spinal cords, we isolated tissue from this region from E12.5 control and *Dot1l*-cKO littermates and performed RNA-seq. In total, we detected 864 transcripts with increased and 647 with decreased expression, applying a cutoff for adjusted p-value below 0.05 (Fig. 1c). A GO enrichment analysis of genes that increased upon loss of *Dot1l* indicated that neuronal differentiation was a process significantly affected in mutant spinal cords. In contrast, genes that decreased upon *Dot1l*-cKO associated amongst others with cell division (Fig. 1d). We analyzed whether altered gene expression correlated with specific progenitor domains or stem cell classes by intersecting this RNA-seq data characterizing *Dot1l*-cKO with genes that were differentially expressed in spinal cord progenitor domains as revealed by single cell RNA-seq [﻿69﻿]. Clustering of differentially expressed genes (DEG) after *Dot1l*-cKO confirmed decreased expression of genes active in progenitors (e.g. *Pax3*, *Bmpr1b*, *Gli3, Jag1, Zic1*), whereas expression of genes involved in differentiation (e.g. *Npy*, *Mafb*, *Lmx1b, Slc6a11*) increased upon loss of *Dot1l* (Fig. 1e). Further, this intersection of the DEG after *Dot1l*-cKO with the single cell RNA-seq data allowed assessing whether specific expression domains, i.e. dorsal or ventral progenitor populations, were affected through *Dot1l* deficiency. This analysis of the intersected DEG revealed that upon *Dot1l*-cKO expression of genes characteristic for dorsal progenitor populations decreased (Fig. 1e, *Pax3, Bmpr1b*). We also observed a fraction of genes with increased expression that are characteristic for ventral genes (Fig. 1e, *Mafb, Lmo1, Grrp1*). We also analyzed the RNA-seq data set of *Dot1l*-cKO for specific marker genes and found significantly decreased expression of *Lhx2* and *Isl1*, marking a dI1 and dI3 subpopulation, respectively. The dI5 interneuron marker *Lmx1b* significantly increased in expression upon *Dot1l*-cKO (Fig. 1f).

Altogether, our observations from the *Dot1l*-cKO spinal cord indicated that 1) neuronal differentiation of spinal cord progenitors might be increased compared to wild type littermates, and 2) mainly the generation of dorsal postmitotic interneurons of different subclasses might be altered.

### DOT1L deficiency reduces expression of interneuron markers in the dorsal spinal cord

**Fig. 2:**
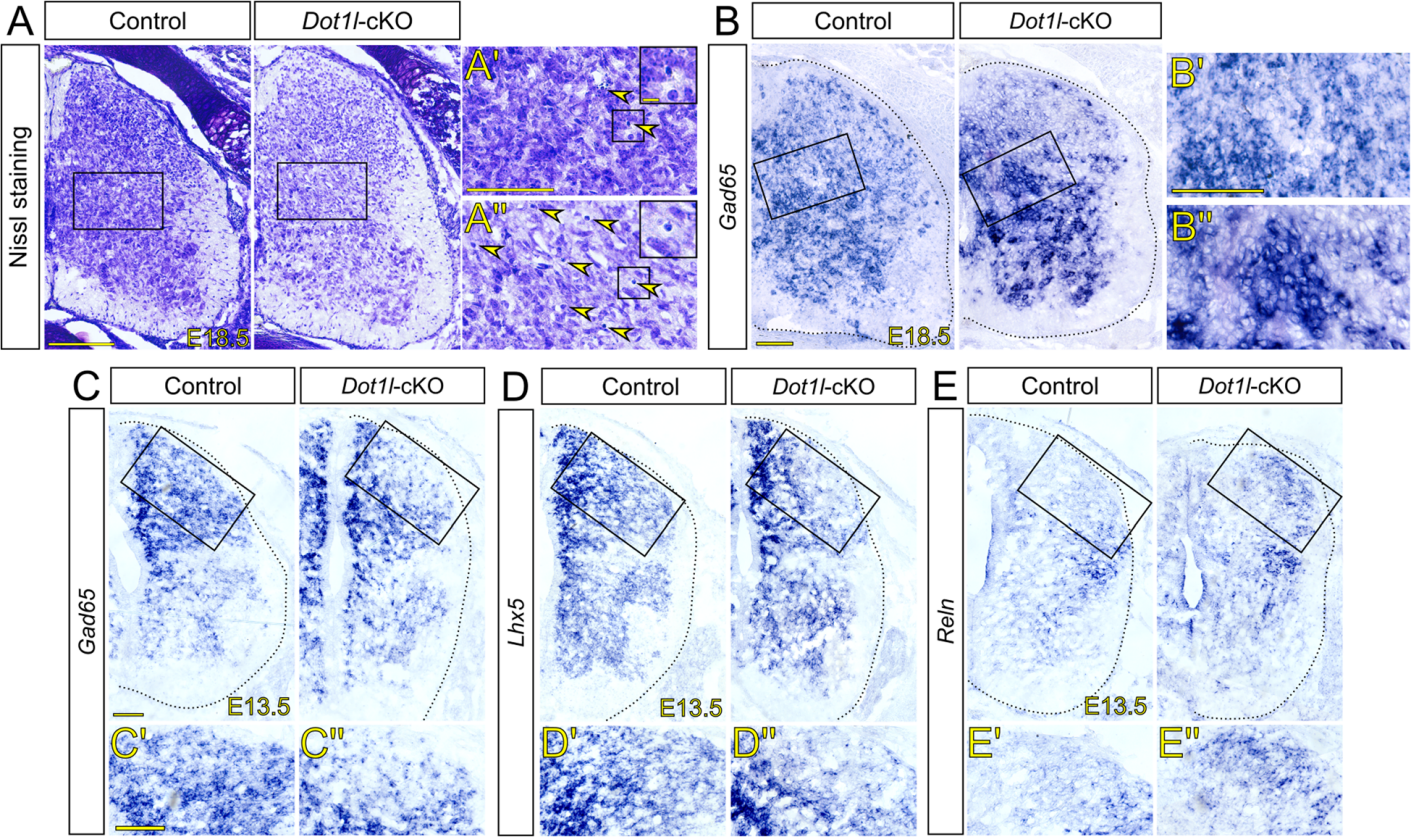
*Dot1l*-cKO causes loss of dorsal differentiation markers at E13.5 and extensive cell death at E18.5. (**a**) Nissl staining on prenatal (E18.5) lumbar spinal cord hemi-sections in control and *Dot1l*-cKO littermates. Black squares in the left panels refer to the higher magnifications, A’ and A” to the right. Within the right panels, boxed magnifications on top right refer to the smaller boxed areas showing pyknotic nuclei. Yellow arrowheads in panels to the right highlight pyknotic nuclei. (**b**, **c**) In situ hybridization (ISH) for *Gad65* transcripts in lumbar spinal cord hemi-sections at E18.5 (**b**) and at E13.5 (**c**) in control and in *Dot1l*-cKO littermates. Higher magnifications in B′, B″, C′, C″. (**d**) ISH for *Lhx5* transcripts in lumbar spinal cord hemi-sections at E13.5 of control and *Dot1l*-cKO. Higher magnifications in D’ and D”, scale as in C, C′. (**e**) ISH for *Reln* transcripts in lumbar spinal cord hemi-sections at E13.5 of control and *Dot1l*-cKO. Higher magnifications in E’ and E”, scale as in C, C′. Black squares: regions of interest described in the results, magnified next or below the figure of reference. Hemicord profiles highlighted by dotted black line. Scale bars: A: 200 μm; A’, B, B′, C, C′: 100 μm; inset in A’: 10 μm

To describe DOT1L function with regard to postmitotic interneurons in the spinal cord, we characterized the *Dot1l*-cKO phenotype at later developmental stages. The *Dot1l*-cKO was lethal in early postnatal stages (Figure S2B), therefore we analyzed the phenotype at E18.5 as the most mature stage. Nissl staining of E18.5 lumbar areas revealed decreased histological staining intensity of the spinal cords for the *Dot1l*-cKO compared to controls (Fig. 2a). Pyknotic nuclei were identified in seemingly higher frequency in mutants compared to controls, mostly concentrated in Rexed lamina VII (Fig. 2a’, a”), supporting the interpretation that less intense staining might be a consequence of increased cell death. Increased numbers of apoptotic cells at E18.5, but not at earlier developmental stages, were confirmed by immunostaining for cleaved CASP3 and through quantification of pyknotic nuclei (Figure S3A-I).

As inhibitory interneurons are the most represented cell type in the spinal cord, we used in situ hybridization (ISH) for *Gad65* to assess generally the patterning and organization of the spinal cord (Fig. 2b). We observed altered expression patterns of *Gad65* within the same area presenting evident cell depletion (Figs. 2a, S3A) in *Dot1l*-cKO compared to controls (Fig. 2b’, b″). *Gad65*-expressing inhibitory interneurons lost their salt-and-pepper patterning within the lumbar area upon *Dot1l-*cKO, but rather appeared in cell clusters with more or less intense staining. This altered expression pattern at E18.5 in *Dot1l-*cKO supported our hypothesis, based on transcriptional changes, that DOT1L might be necessary for proper interneuron differentiation of various subtypes. Further, these results suggested that derailed interneuron differentiation and positioning might cause increased rates of dying cells in later, i.e. E18.5, compared to earlier, i.e. E11.5-E13.5, developmental stages.

To characterize subpopulations of interneurons at earlier stages, we analyzed *Gad65*, *Lhx5* and *Reln* expression at E13.5, at the end of neurogenesis. At E13.5 *Gad65* expression defines functionally the emerging classes of dI4, dI6, V1 and V2b inhibitory interneurons [68]. *Dot1l*-cKO produced a reduction of *Gad65*-transcripts in the dorsal mantle area compared to control littermates (Fig. 2c, c’ and c″ respectively control and *Dot1l*-cKO). *Lhx5* molecularly defines dI2, dI4, dIL_A_, dI6, V0 and V1 [17]. *Lhx5-*expressing cells distributed similarly as *Gad65*-expressing cells, with decreased signal intensity in the dorsal mantle zone of mutant compared to control embryos (Fig. 2d, d’, d”). *Reln* transcription marks V1 and V2 interneurons [70, 71]. *Dot1l*-cKO spinal cords showed ectopic expression of *Reln* in dorsal areas (Fig. 2e, e’, e”). Altogether, expression analysis of broadly expressed interneuron markers suggested either that interneuron populations from dorsally located progenitors decreased in *Dot1l*-cKO or that they migrated ventrally. In contrast to this observation, the results suggested that *Reln*-expressing ventral interneurons might have invaded from ventral into the dorsal area or that *Reln* was ectopically expressed.

### DOT1L is necessary for proper localization of dorsal and ventral interneuron populations

**Fig. 3:**
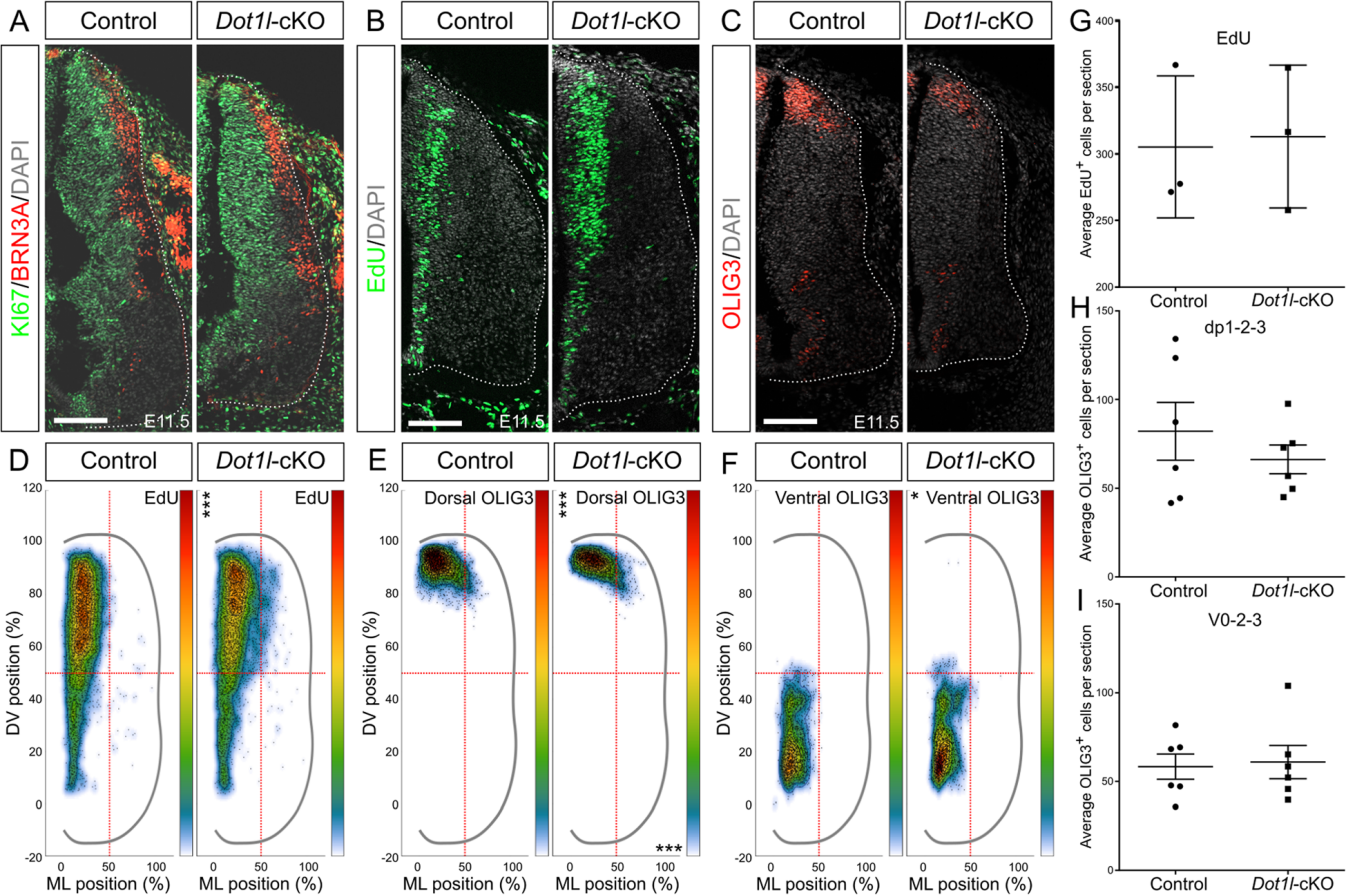
*Dot1l*-cKO results in defective early differentiation and migration of interneurons without affecting progenitor proliferation. (**a**) Immunostaining for KI67 (green), BRN3A (red) and DAPI (gray) on lumbar spinal cord hemi-sections at E11.5 from control and mutant littermates. (**b**) Representative images of immunostaining of EdU (green, 30 min pulse) and DAPI (gray) on E12.5 lumbar spinal cords in control and *Dot1l*-cKO embryos. (**c**) Representative images of immunostaining for OLIG3 (red) and DAPI (gray) on lumbar spinal cord hemi-sections at E11.5 from control and mutant littermates. Scale bars: 100 μm. (**d, e, f**) Density plots from EdU (**d**) and OLIG3 (**e** dorsal, **f** ventral interneuron). Density plot projections were analyzed by multivariate analysis for Hotelling’s two-sample square test; * *p* < 0.05, *** *p* < 0.001, stars are reported on the Y axis (dorsoventral, DV) or X axis (mediolateral, ML) according to values on the individual axes. (**g, h, i**) Quantitative analysis performed for EdU (**g**), dorsal OLIG3 interneurons (**h**) and ventral OLIG3 interneurons (**i**). Quantifications represented with mean ± SEM. P-values were calculated with unpaired, two-tailed Student’s *t*-test. Per staining, 4 hemi-sections were counted for each n (EdU *n =* 3, OLIG3 *n* = 6 both dorsal and ventral). Hemicord profiles highlighted by dotted white line. Scale bars: 100 μm

To investigate the development of different interneuron classes upon *Dot1l*-cKO in greater detail, we first assessed cell proliferation at E11.5 using immunofluorescence of the general cell cycle marker KI67 together with BRN3A, which demarcated multiple dorsal differentiating neuronal populations (dI1-3, dI5 and dIL_B_) (Fig. 3a). Both markers did not reveal obvious differences between *Dot1l-*cKO and control animals. Similarly, a comparable fraction of progenitor cells in the ventricular zone was observed using immunostainings against SOX2 (Figure S2C). We labeled cycling progenitors with a 30 min EdU pulse and quantified cells that incorporated EdU. We observed a significant dorsoventral shift of EdU-positive cells in the lower dorsal area of mutant compared to control littermates, despite unchanged total numbers of EdU-positive cells (Fig. 3b, d, g). EdU-positive cells populated a large dorsal domain that extended into the mantle zone suggesting accelerated exit from the cell cycle and differentiation of dorsal interneurons. OLIG3-expressing dorsal progenitors dp1-2-3 and early postmitotic interneurons dI1-2-3 [8] also displaced from the ventricular zone at E11.5, and the OLIG3-expression domain extended towards the mantle zone (Fig. 3c). This dorsal OLIG3-expressing progenitor and early postmitotic cell population did not change in total number between genotypes (Fig. 3h). We therefore concluded that *Dot1l*-cKO affected migration and differentiation of specific dorsal interneuron populations rather than proliferation of progenitors residing in the dorsal ventricular zone.

Ventral postmitotic interneurons express *Olig3* as well [72]. Analysis of OLIG3-expressing cells in the ventral domain (Fig. 3c, f) indicated a slight shift along the dorsoventral axis and increased ventral density upon loss of DOT1L. Similar to the dorsal, we did not observe a significant change in cell numbers in the ventral OLIG3 population (Fig. 3h, i). We concluded that loss of DOT1L affected distribution and hence early migration mainly but not exclusively of dorsal OLIG3 interneuron subpopulations. This observation was also in accordance to stable transcriptional levels of *Olig3* upon *Dot1l*-cKO (Fig. 1f). OLIG3 expression is an early hallmark of dI1-2-3 populations. Therefore, we further characterized systematically these dorsal interneuron populations with regard to marker gene expressions and their quantitative or qualitative changes.

### DOT1L deficiency reduces numbers of LHX2- and shifts LHX9-expressing dI1 interneurons to a lateroventral position

**Fig. 4:**
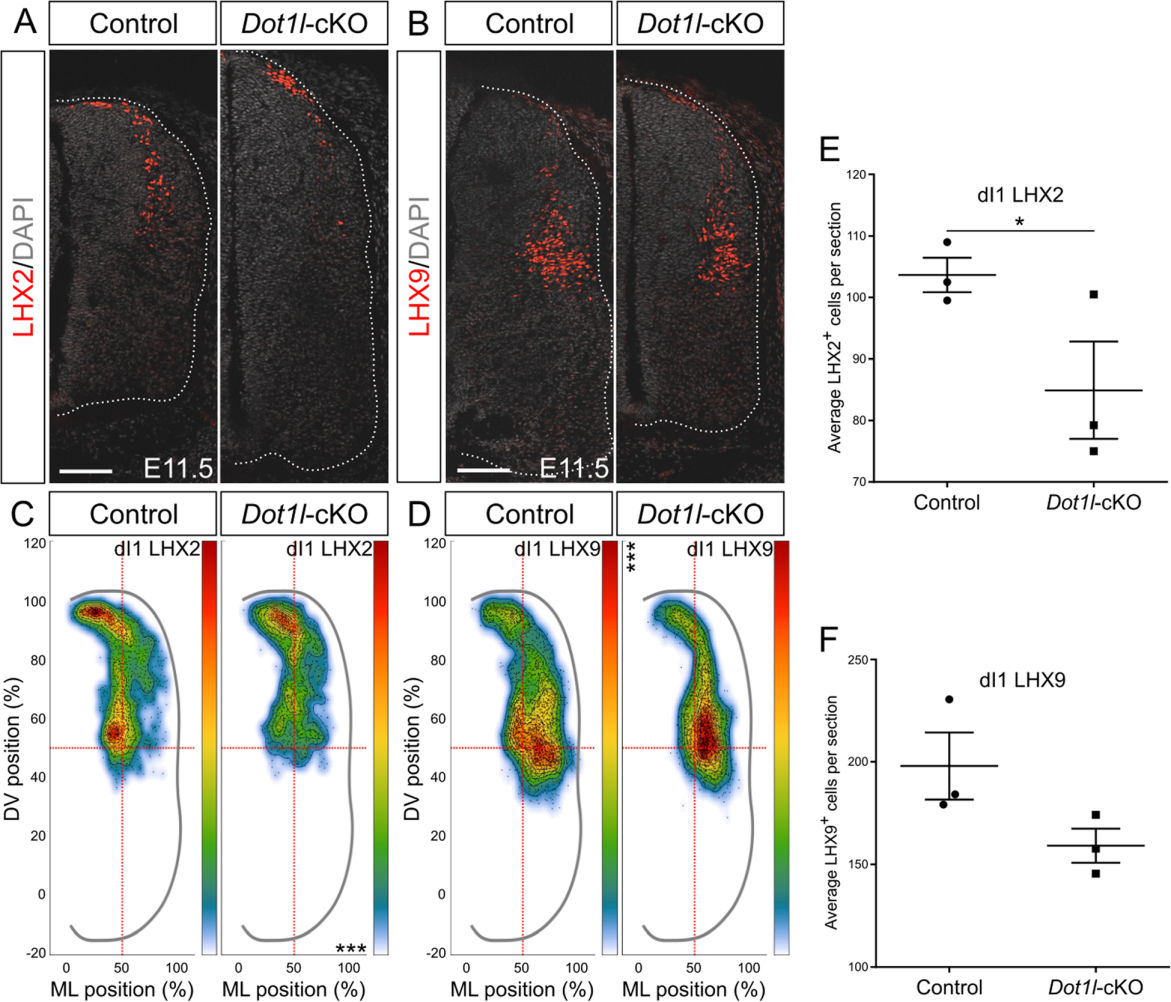
*Dot1l*-cKO decreases LHX2-dI1 interneurons and derails migration of the LHX9-dI1 interneurons at E11.5. (**a, b**) Representative immunostainings for LHX2 (red) and DAPI (gray) in (**a**) or LHX9 (red) and DAPI (gray) in (**b**) on spinal cord hemi-sections of control and mutant littermates at E11.5. Hemicord profiles highlighted by dotted white line. Scale bars: 100 μm. (**c, d**) Density plots from LHX2 (**c**) and LHX9 (**d**). Density plot projections were analyzed by multivariate analysis for Hotelling’s two-sample square test; *** *p* < 0.001, stars are reported on the Y axis (dorsoventral, DV) or X axis (mediolateral, ML) according to values on the individual axes. (**e, f**) Quantitative analyses of immunostainings for LHX2 (**e**) and LHX9 (**f**). Quantifications represented with mean ± SEM. P-values were calculated with unpaired, one-tailed (**e**) or two-tailed (**f**) Student’s *t*-test, * *p* < 0.05. Per staining, 4 hemi-sections were counted for each n (*n* = 3)

The dorsal-most interneuron class, dI1, is characterized by expression of *Lhx2* and *Lhx9*. Expression of both markers changes dynamically throughout neurogenesis. From a shared pool of progenitors, two subpopulations emerge based on the proportion of *Lhx2* and *Lhx9* transcripts: ventromedially located interneurons express highly LHX2 and to a lesser extend LHX9, whereas a ventrolateral counterpart expresses highly LHX9 but not LHX2 [73]. Intersection of DEG upon *Dot1l*-cKO with the genes having characteristic expression in specific domains of the spinal cord [68] revealed that *Lhx2* was present in the intersected genes and decreased in transcription upon loss of DOT1L as revealed by RNA-seq (Figs. 1f, S5A). *Lhx9* transcript was not differentially expressed in *Dot1l*-cKO embryos. Based on this observation, we analyzed separately the subsets of the dI1 population using immunostainings. At E11.5, the LHX2-positive subpopulation was significantly reduced upon loss of DOT1L, particularly in its ventral-most subset, whereas control littermates consistently displayed cells in a continuous migratory stream towards the intermediate ventral area (Fig. 4a, c, e). The quantification of the LHX9-expressing subpopulation revealed that the decreased number of LHX2-expressing dI1 precursors was not accompanied by a significant concurrent decrease or a compensatory increase in cells expressing LHX9 (Fig. 4b, d, f). Although *Dot1l*-cKO did not change the LHX9-expressing subpopulation quantitatively compared to controls, the distribution of these interneurons was more restricted on the mediolateral axis. Furthermore, LHX9-positive dI1 interneurons distributed with a significantly increased density in the mediolateral spinal cord (Fig. 4d). This area is the target region of ipsilaterally projecting dI1 interneurons highly expressing LHX9 [73].

**Fig. 5:**
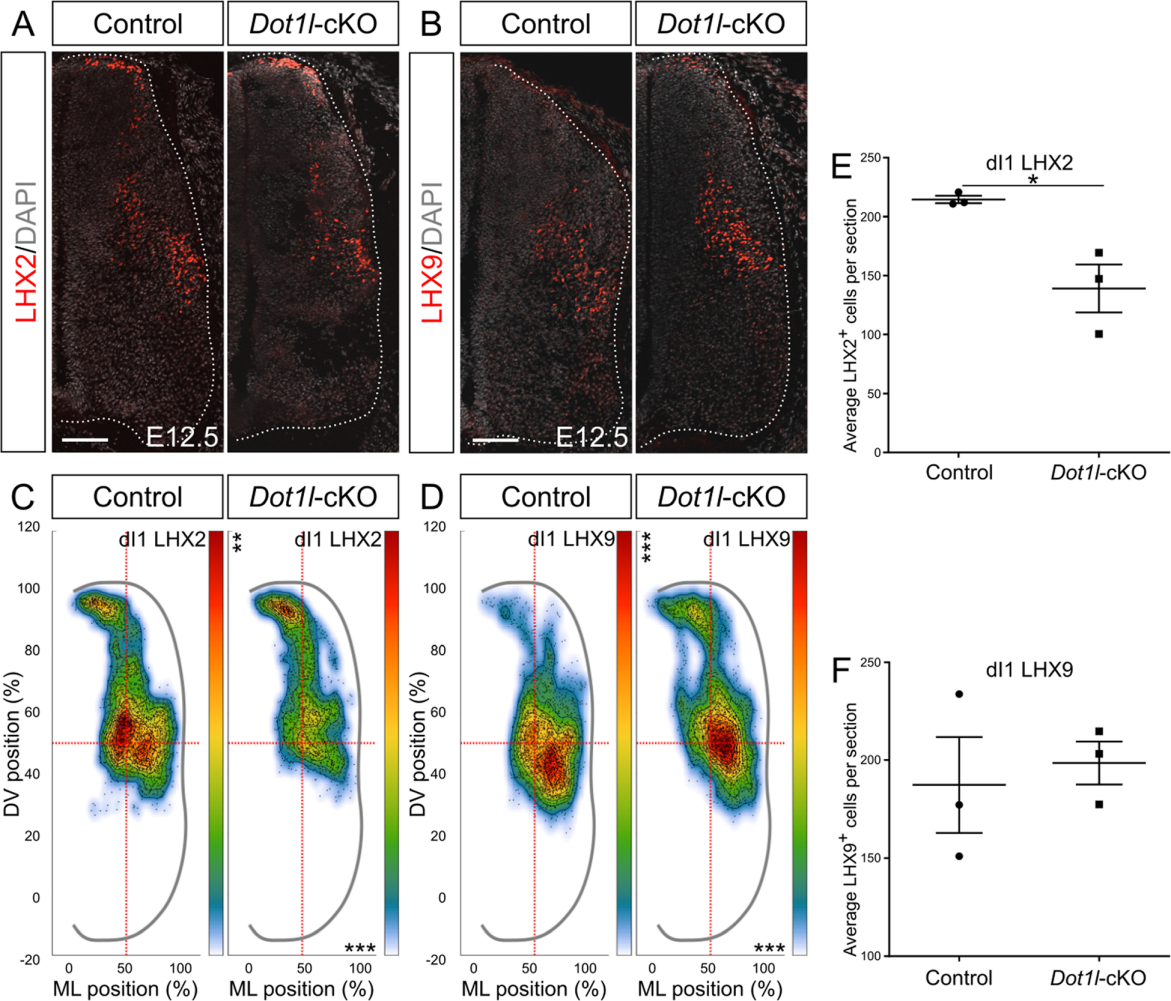
*Dot1l*-cKO decreases LHX2-dI1 interneurons and derails migration of the LHX9-dI1 interneurons at E12.5. (**a, b**) Representative immunostainings for LHX2 (red) and DAPI (gray) in (**a**), and LHX9 (red) and DAPI (gray) in (**b**) on spinal cord hemi-sections of control and mutant littermates at E12.5. Hemicord profiles highlighted by dotted white line. Scale bars: 100 μm. (**c, d**) Density plots from LHX2 (**c**) and LHX9 (**d**). Density plot projections were analyzed by multivariate analysis for Hotelling’s two-sample square test; *** p < 0.001, stars are reported on the Y axis (dorsoventral, DV) or X axis (mediolateral, ML) according to values on the individual axes. (**e, f**) Quantitative analyses of immunostainings for LHX2 (**e**) and LHX9 (**f**). Quantifications represented with mean ± SEM. P-values were calculated with unpaired, one-tailed (**e**) or two-tailed (**f**) Student’s *t*-test; * *p* < 0.05. Per staining, 4 hemi-sections were counted for each n (*n* = 3)

Further on in development at E12.5, the total cell number of LHX2-expressing dI1 cells was still reduced in *Dot1l*-cKO (Fig. 5a, e). Mostly the cell population in ventral positions contributed to the quantitative differences (Fig. 5c). We did not observe an evident increase in cell death that could account for the quantitative differences (Figure S3C-H). In further accordance to our observation at E11.5, the LHX9 dI1 cells did not change significantly in cell numbers in mutant compared to controls at E12.5 (Fig. 5b, f). However, the LHX9 dI1 subpopulation accumulated significantly denser at mediolateral positions, similar to the earlier developmental stage (Fig. 5d). In summary, the two dI1 subpopulations behaved differently upon *Dot1l*-cKO, as the LHX2-positive subset decreased upon cKO while LHX9 cells were unaffected in numbers but occupied majorly and more densely their target area. These results were in line with the transcriptomic observations of a selective decrease of *Lhx2* expression without differential expression of *Lhx9*, suggesting that the LHX2-expressing subset depends on DOT1L presence for its identifier marker expression and molecular identity. On the other hand, DOT1L affects proper localization of the LHX9-expressing subset.

### DOT1L affects localization of dI2 interneurons

**Fig. 6:**
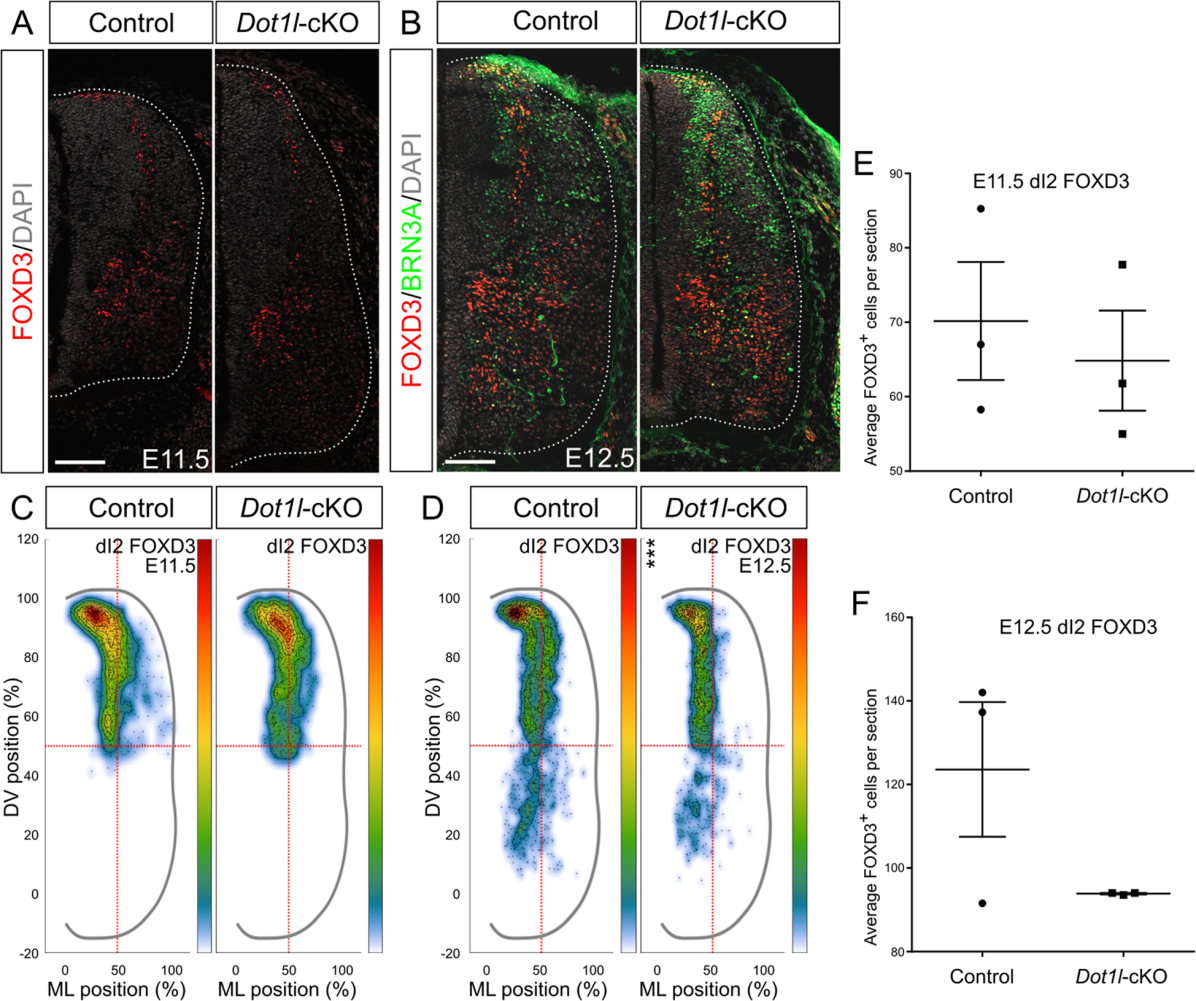
*Dot1l*-cKO impairs the dI2 migration path. (**a, b**) Representative immunostainings for dI2 and V1 interneurons at E11.5 (**a**) and E12.5 (**b**). In (**a**), dorsal FOXD3 (red) labels dI2 interneurons and ventral FOXD3 marks V1, while DAPI (gray) stains all the nuclei. In (**b**), dI2 are dorsally marked by FOXD3 (red) and ventrally by the costaining of FOXD3 and BRN3A (green). V1 in (**b**) are ventral cells labeled uniquely by FOXD3. Hemicord profiles highlighted by dotted white line. Scale bars: 100 μm. (**c, d**) Density plots for dI2 interneurons at E11.5 (**c**) and E12.5 (**d**). Density plot projections were analyzed by multivariate analysis for Hotelling’s two-sample square test; *** p < 0.001. Stars are reported on the Y axis (dorsoventral, DV) or X axis (mediolateral, ML) according to values on the individual axes. (**e, f**) Quantitative analyses of immunostainings for dI2 at E11.5 (**e**) and E12.5 (**f**). Quantifications represented with mean ± SEM. P-values were calculated with unpaired, two-tailed Student’s *t*-test. Per staining, 4 hemi-sections were counted for each n (*n* = 3)

Similarly to dI1, the dI2 subclass initially expresses OLIG3 and migrates to ventral positions [74]. We performed a staining for FOXD3, marking both dI2 and V1 domains at E11.5 (Fig. 6a) and E12.5 (Fig. 6b). We did not observe significant alterations in cell numbers or variation in distribution for FOXD3-expressing dI2 interneurons at E11.5 (Fig. 6c, e). At E12.5, we co-stained for FOXD3 and BRN3A to detect the entire dI2 population (Fig. 6b), since migrating dI2 interneurons temporarily lose BRN3A and maintain FOXD3 expression, but they re-express BRN3A when reaching their target medial area. At this later stage, FOXD3 cells presented a dorsoventral position shift upon *Dot1l*-cKO (Fig. 6d), despite cell numbers that were similar compared to control littermates (Fig. 6f). Indeed, the ventral area, which is the final destination of this neuronal subpopulation, was populated by fewer dI2 interneurons in mutants compared to controls (Fig. 6d), suggesting a migratory delay for the overall dI2 pool in the *Dot1l*-cKO in comparison to control littermates. Together these experiments showed a role of DOT1L in the development and differentiation of dI2 interneurons, upon which DOT1L influenced most likely their migration.

### DOT1L deficiency shifts dI3 interneurons to ventral positions during the course of development

**Fig. 7:**
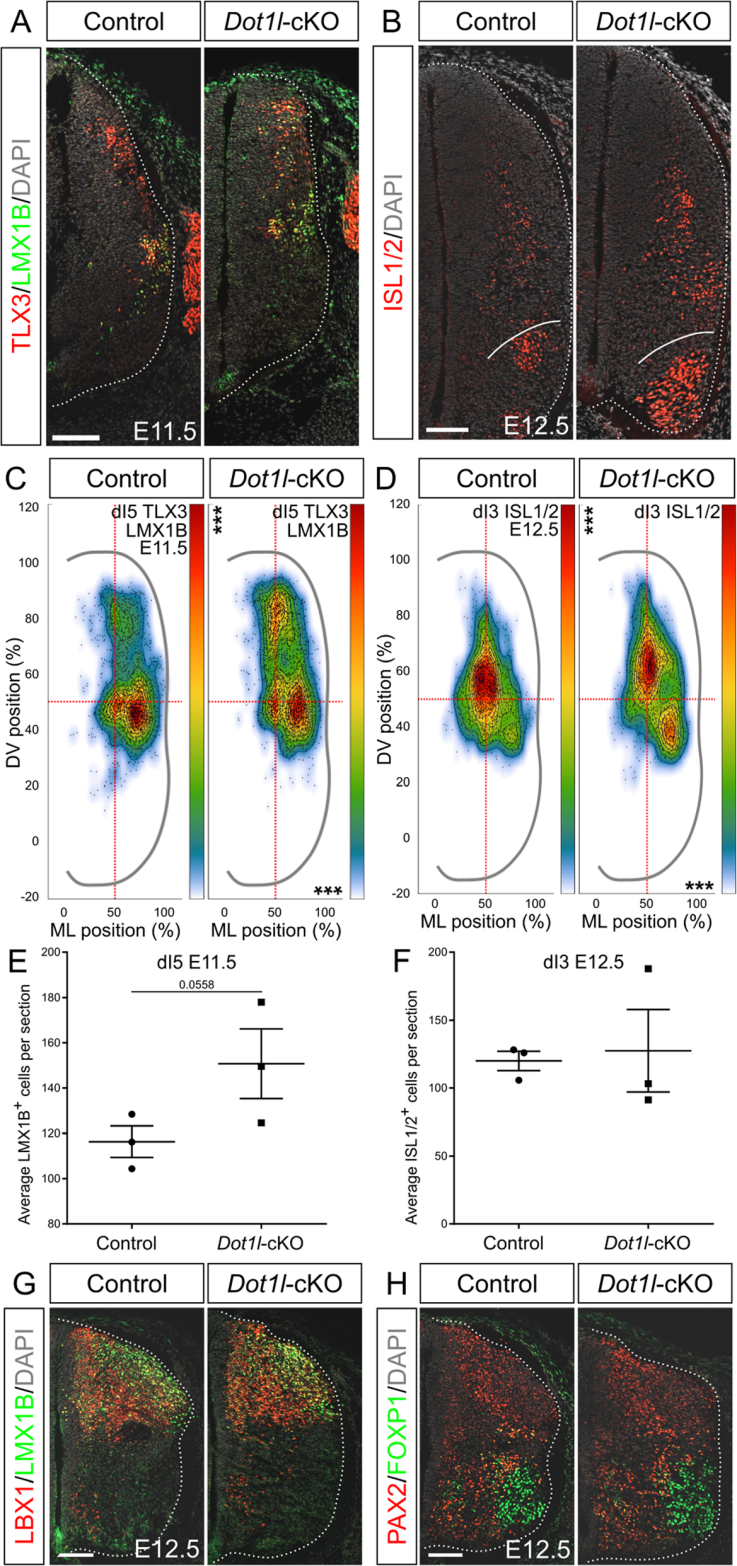
DOT1L differentially controls dI5 early positioning and dI3 late distribution. (**a**) Representative immunostainings for dI3 and dI5 interneurons at E11.5. dI3 interneurons are labeled by TLX3 staining (red) and dI5 are labeled by both TLX3 and LMX1B (green), while DAPI (gray) stains all the nuclei. (**b**) Representative immunostainings for dI3 at E12.5. dI3 interneurons are labeled by ISL1/2 staining (red), while DAPI (gray) counterstains all the nuclei. (**c, d**) Density plots for dI5 interneurons at E11.5 (**c**) and dI3 interneurons at E12.5 (**d**). Density plot projections were analyzed by multivariate analysis for Hotelling’s two-sample square test; *** p < 0.001. Stars are reported on the Y axis (dorsoventral, DV) or X axis (mediolateral, ML) according to values on the individual axes. (**e, f**) Quantitative analyses of immunostainings for dI5 at E11.5 (**e**) and dI3 at E12.5 (**f**). (**g, h**) Representative immunostainings of LBX1 (red) and LMX1B (green) for excitatory interneurons (**g**) and PAX2 (red) in costaining with FOXP1 (green) for inhibitory interneurons (**h**) at E12.5. Hemicord profiles highlighted by dotted white line. Scale bars: 100 μm. Quantifications represented with mean ± SEM. P-values were calculated with unpaired, two-tailed Student’s *t*-test. Per staining, 4 hemi-sections were counted for each n (*n* = 3)

The third dorsal class marked by OLIG3 is dI3, a subpopulation that shares excitatory fate with the more ventrally localized dI5 interneurons [75, 76]. Both dI3 and dI5 excitatory interneurons express TLX3 as fate determinant. dI3 and dI5 interneurons use different migratory paths, where dI3 migrates ventrally, while dI5 settles in the dorsal horn [2]. To track the two populations we co-stained lumbar sections at E11.5 for TLX3 and LMX1B (Fig. 7a) and quantified on the basis of TLX3-single (dI3) or TLX3/LMX1B-co-expressing (dI5) cells. The dI3 cell population showed neither statistically significant positional nor quantitative changes in mutant littermates compared to control animals at E11.5 (Figure S4A, B). At E12.5, we studied the development of dI3 through immunostaining of ISL1/2 cells, labeling dI3 and MN (Fig. 7b). Whereas the total cell numbers were unchanged (Fig. 7f), the dI3 populations differed among conditions in their distribution (Fig. 7d). In control littermates, dI3 interneurons appeared as one uniform and intermediate population in the dorsal mantle zone. Conversely, the region populated by dI3 interneurons in *Dot1l*-cKO split into two hotspot regions. These results supported the conclusion that early dI3 interneurons do not contribute to the shift in the OLIG3 pool upon *Dot1l*-cKO, but that at later stages dI3 interneuron migration is altered by loss of DOT1L.

### DOT1L deficiency shifts dI5 interneurons to dorsal positions

At E11.5, distributional analysis of cells co-expressing TLX3 and LMX1B highlighted increased density of dI5 cells in the dorsal area, towards the developing dorsal horn upon *Dot1l*-cKO (Fig. 7c). This observation suggested that dI5 excitatory interneurons would reach their respective target region earlier upon DOT1L deletion compared to control animals. In addition, we observed that the quantitative changes upon DOT1L deletion were close to significance which might point towards increased numbers of cells expressing LMX1B (Fig. 7e). To get further evidence corroborating a putative quantitative change in dI5 cells, we inspected intersected DEG upon *Dot1l-*cKO from the RNA-seq analysis with domain-specific differentiation genes, two datasets that were generated at the slightly later stage E12.5 (Figs. 1f, S5A). We observed that *Lmx1b* transcription increased upon loss of DOT1L (adjusted p-value below 0.005, log_2_FC of 0.315). Similarly, the transcript for *Phox2a*, which is a known marker for a subset of dI5 interneurons [77], increased significantly in *Dot1l*-cKO samples (adjusted p-value below 0.05, log_2_FC of 0.377). Together, increased levels of *Lmx1b* and *Phox2a* supported the view that dI5 subpopulations might be expanded in mutant compared to control littermates. To describe the dI5 subpopulation at E12.5, we performed immunostainings for LMX1B excitatory interneurons (Fig. 7g). In contrast to the increased transcription of *Lmx1b* within the E12.5 transcriptome, no evident phenotype upon DOT1L depletion was detected using immunostainings at E12.5. We concluded that the dI5 subpopulation of interneurons might not be disturbed in generation and localization upon *Dot1l-*cKO.

### DOT1L does not affect development of dI4 interneurons

The expression of the general inhibitory interneuron marker *Gad65* at E13.5 suggested a reduction of inhibitory interneurons, which derive from the dI4 subclass [6, 68] in the dorsal horn (Fig. 2c). We therefore performed immunostainings at E12.5 for LBX1 to further characterize dI4 and dI6 interneuron domains (LBX1 single-labeled) flanking dI5 (co-labeled for LMX1B and LBX1 in Fig. 7g). In addition, we also assessed PAX2 expression to study the development of dI4 and dI6 towards their inhibitory fate (Fig. 7h). Although we observed at E13.5 a less intense staining pattern for *Gad65* and *Lhx5* inhibitory interneurons (Fig. 2c, d) we could not identify a marked phenotype on E12.5 of the inhibitory dI4 subpopulation in the developing dorsal horn upon DOT1L deletion.

### DOT1L deficiency shifts EVX1-expressing V0 interneurons to a mediolateral position

**Fig. 8:**
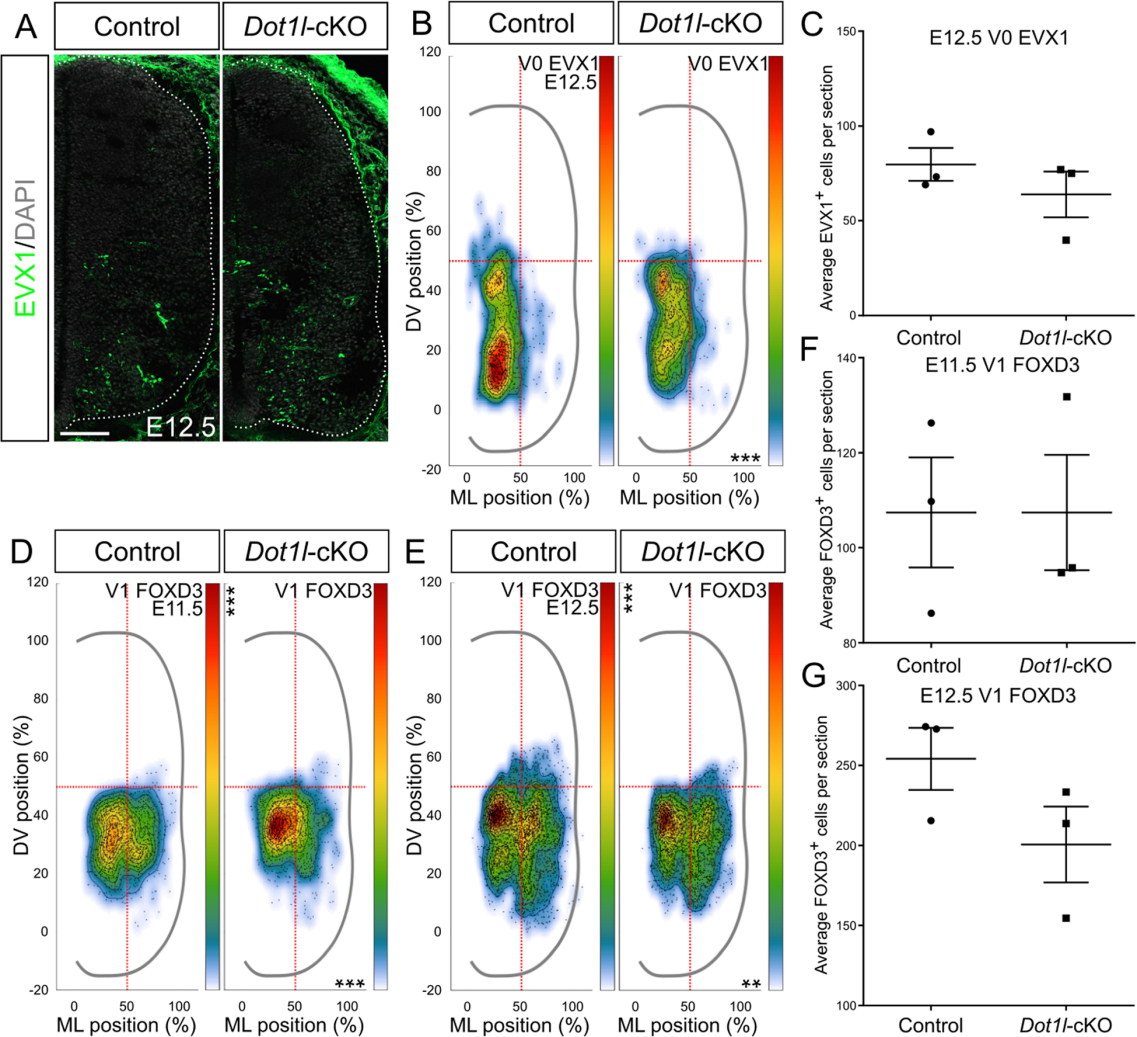
DOT1L depletion at E12.5 causes distributional defects of V0 and reduces V1 migration. (**a**) Representative immunostainings for EVX1 (green) and DAPI (gray) on spinal cord hemi-sections of control and mutant littermates at E12.5. Hemicord profiles highlighted by dotted white line. Scale bars: 100 μm. (**b**) Density plots from EVX1-expressing cells. (**c**) Quantitative analysis of EVX1, represented with mean ± SEM. (**d, e**) Density plots for V1 interneurons from Fig. 6a (E11.5, ventral cells FOXD3-expressing) and 6b (E12.5, ventral cells FOXD3 single-labeled), respectively at E11.5 (**d**) and E12.5 (**e**). Density plot projections were analyzed by multivariate analysis for Hotelling’s two-sample square test; ** p < 0.005, *** p < 0.001, stars are reported on the Y axis (dorsoventral, DV) or X axis (mediolateral, ML) according to values on the individual axes. (**f, g**) Quantitative analyses of immunostainings for FOXD3-positive V1 at E11.5 (**f**) and E12.5 (**g**). P-values were calculated with unpaired, two-tailed Student’s *t*-test. 4 hemi-sections were counted for each n (*n* = 3)

As our analysis of the OLIG3-expressing progenitors at E11.5 suggested that V0 interneurons could also be affected by loss of DOT1L, we used EVX1 expression as further marker for a subset (V0_V_ and V0_CG_) of V0 interneurons (Fig. 8a) [78, 79]. We did not detect changes in cell number at E12.5 (Fig. 8c), but a slight mediolateral shift in the position of the expression domain coupled with a more homogeneous cell density distribution in *Dot1l-*deficient compared to control littermates (Fig. 8b). We therefore concluded that the V0 subpopulation did not contribute to the denser distribution of OLIG3-expressing progenitors in the ventral spinal cord at E11.5 (Fig. 3f). However *Dot1l-*cKO affected the localization of the V0 population, which presented an altered migration towards the ventral positions, where the mutant cells occupied lateral domains within the migratory path compared to control interneurons. This distribution of the V0 cells suggested to us that loss of DOT1L delays generally V0 interneuron migration.

### DOT1L deficiency mildly redistributes V1 interneurons in ventral areas

Due to the observed abnormal localization of *Reln* transcripts at E13.5 (Fig. 2e), we also investigated V1 interneuron development and positioning. At E11.5, cells expressing FOXD3 in the ventral spinal cord did not show changes in the cell numbers (Fig. 8f), but again we observed a significant shift in cell density in mutant compared to control littermates (Figs. 6a, 8d), showing a less expanded domain populated by V1 interneurons in mutant littermates. The population size of single FOXD3-expressing ventral cells did not change as well at E12.5 (Figs. 6b, 8e). Coherently with E11.5, V1 interneurons distributed in a less extended area in mutants compared to controls (Fig. 8g). At the time points studied, V1 cells occupied a smaller area in mutant littermates, displaying migration delay based on cell position. We did not observe evidence of a derailed migration and population of V1 interneurons towards the developing dorsal horn, suggesting that the dorsally located cells ectopically expressed *Reln* in *Dot1l*-cKO animals.

## Discussion

The data reported here showed that DOT1L is a chromatin modifier implicated in transcriptional control during spinal cord development. Specifically, dorsal activity of DOT1L was required for proper development of dI1 interneurons. In this context, DOT1L functions were pleiotropic, because loss of DOT1L (through Wnt1-cre) reduced the number of the LHX2-expressing dI1 subclass within the dorsal horn, whereas the LHX9-expressing subclass of dI1 interneurons displayed rather a migratory or positioning defect in the target area. Investigation of other interneuron populations in the dorsal spinal cord (dI2, dI3, dI4 and dI5) and in selected ventral domains (V0 and V1) indicated that the *Dot1l*-cKO led to aberrant localization for most of the developing interneuron classes that we analyzed. Aberrant interneuron positioning took place during the neurogenesis waves around E11.5 and E12.5. These early events likely contributed to the derailed patterning observed also at the last stage this mouse line could be analyzed, at E18.5. We hypothesize that DOT1L contributes via transcriptional regulation of genes involved in cell migration and differentiation to correct specification and patterning of the spinal cord. We assume that the defective positioning and deregulation of cues for cell migration observed during initial phases of spinal cord development manifest later as altered regionalization and/or neuronal network composition. Increased cell death at the late developmental stage, i.e. E18.5, might be a consequence of this mis-patterning of the spinal cord after *Dot1l-*cKO, because we did not observe that DOT1L deficiency increased apoptosis during early stages, i.e. E11.5-E13.5. As late prenatal stages physiologically present apoptotic waves, the larger cell death rate upon *Dot1l*-cKO could be a direct consequence of a defective network of cells misplaced in comparison to their physiological environments.

### DOT1L-cKO using Wnt1-cre does not affect early neurulation

Our study to investigate DOT1L function in spinal cord development was motivated by recent studies, which correlated epigenetic modifications with the onset of neural tube defects [36, 37, 78]. Moreover, a direct link that DOT1L might be necessary for proper spinal cord development was suggested through observation that lack of H3K79 dimethylation, mediated by DOT1L activity, occurred at higher frequency in human fetuses presenting with spina bifida [43]. In chicken embryos, H3K79 homocysteinylation in place of methylation associated with increased NTD [44]. However, although we observed a slight increase of NTD in chicken exposed to an inhibitor that pharmacologically blocked DOT1L activity, the total number of neurulation defects was low (ranging from 13 to 26% after DOT1L inhibition and 0 to 5% in controls at E3 or E3.5). Employing a conditional mouse model by using Wnt1-cre as driver line neither produced observable neurulation defects, similarly to a previously characterized Atoh-cre driven *Dot1l*-cKO [55], nor resulted in an obvious phenotype within progenitor cells at earlier stages (Figure S2C-G). Thus, our phenotypic characterizations of DOT1L inactivation with the specific Wnt1-cre or Atoh-cre driver mouse lines do not support an association between neurulation defects and reduction of H3K79me2 in the dorsal part of the developing spinal cord. It cannot be ruled out, however, that NTD upon DOT1L inactivation occurs if other cre-driver lines are used. Hence, more research is needed in support of an involvement of DOT1L in NTD.

### DOT1L is necessary for dI1 differentiation and interneuron migration

The most striking phenotype we observed in Wnt1-cre *Dot1l*-cKO was an altered distribution of the OLIG3-expressing precursor population that will generate dI1, dI2 and dI3 interneurons. In addition, we observed numerical alterations upon *Dot1l*-cKO within the descendants of the OLIG3 lineage belonging to the dI1 subset expressing LHX2. But interestingly, DOT1L-deficiency did not affect all OLIG3-derived interneuron subpopulations equally. Whereas LHX2-positive progenitors decreased in numbers, LHX9-expressing cells mainly distributed differently and did not display quantitative alteration. *Lhx2* is a direct target, i.e. marked with H3K79me2, of DOT1L in the cerebral cortex. It is therefore tempting to assume that within the OLIG3-lineage *Lhx2* is transcriptionally activated by DOT1L-mediated H3K79me2. Loss of DOT1L would impair *Lhx2* expression and subsequent differentiation of this interneuron cell lineage. The establishment and maintenance of molecular identity necessary for differentiation into the *Lhx9*-expressing interneuron lineage, as well as other subtypes, including dI2 and V0, would be independent of DOT1L activity. The assumption that DOT1L activity is needed for neuronal subtype specification is in accordance with our observations during cerebral cortex development, where DOT1L facilitates differentiation of neurons mainly localized in superficial layers [57].

Another relevant aspect of the observed phenotype following *Dot1l*-cKO is a defective interneuron migration and localization, as for example seen in the LHX9-expressing dI1 subpopulation. Diverse genes participating in the biological processes of neuron migration and axonal projection showed altered expression in DOT1L depleted embryos at E12.5 (Figure S5B, C). Among those, we observed *Sema3b*, *Sema3f*, *Sema6b* and *Plxnb3*, and as well *Epha3*, *Ephb6* and *Epha8*, members of gene families known to be essential for migration and axonal projection in the spinal cord [79–81]. Immunofluorescent analyses confirmed that DOT1L depletion differentially affects interneuron populations, notably resulting in an early phenotype (E11.5) for dI5, V0 and V1 interneurons, and a late phenotype (E12.5) for dI2 and dI3 populations. Within the early phenotype, *Dot1l*-cKO affected interneuron migration/localization differently: whereas mutant dI5 (LMX1B-expressing) interneurons occupied the dorsal horn position earlier than in control littermates, V0 (within the OLIG3 pool) and V1 (ventral FOXD3) interneurons likely presented a migration delay, as the mutant cells failed to reach the physiological location within the ventral areas. Interestingly, although V1 displayed derailed lateral migration, its mispositioning does not support the idea of an invasion of V1 in the dorsal horn. As it is unlikely that ectopic expression of *Reln* in the dorsal horn at E13.5 was thus caused by V1 cells, it is possible that aberrant *Reln* transcription was caused directly by the depletion of DOT1L, as previously described [57].

Upon DOT1L deletion we observed increased cell death within Rexed laminae V-VII only late in development at E18.5, whereas increased apoptosis was absent from early, neurogenic stages upon *Dot1l*-cKO. Therefore, decreased expression of LHX2, *Lhx5* or *Gad65* upon *Dot1l*-cKO was not caused by abnormal cell death. We thus hypothesize that the impaired positioning of interneurons was followed by elimination of misplaced neurons or defective neuronal networks. In addition, the transcriptome analyses suggested that in addition to migratory cues (Figure S5B, different Ephrins and Semaphorins, *Zic2*, *Runx3*, *Plxnb3)* the *Dot1l-*cKO resulted in deregulation of synapse or neuronal network organization (Figure S5D, e.g. *Syn1*, *Shank1*, *Dvl1*, *Neurod2*), all of which might alter the cellular environment into which newly generated interneurons enter and the capability of interneurons to form synapses. As reported, the environment surrounding each interneuron crucially impacts on its survival, which presumably is also supported by functional network integration through direct synaptic connections [82].

Our data from the developing spinal cord and its dependence on DOT1L showed that within this model system DOT1L might not primarily affect survival by directly controlling gene expression programs that lead to apoptosis. This notion is in accordance with our observations from the cerebral cortex, where we only observed activation of ER stress programs in vitro but not in vivo [57, 58]. Cell death was also excluded as cause for phenotypic alterations present after DOT1L deletion in the developing cerebellum [55]. In conclusion, as DOT1L mediated control of cell survival seems restricted to cellular stress under in vitro culture conditions, increased cell death only in later developmental stages in the spinal cord is most likely an indirect effect.

### DOT1L balances progenitor proliferation, differentiation and neuronal migration

Context- or cell-dependent DOT1L functions are obvious from the GO enrichment analysis of DEG after *Dot1l*-cKO in the spinal cord. Here, cell cycle parameters might be negatively affected (i.e. *Anp32b*, *Cdc14a*, *Cdc45*, *Ccne1*, *Cdkn1a*) and differentiation programs were prematurely activated (*Npy*, *Cbln2*, *Mafb*, *Lmx1b*). Although this observation would have been in line with our studies from *Dot1l*-cKO in the developing cerebral cortex and cerebellum, we could not confirm by immunostainings followed by quantifications that DOT1L preserves progenitor properties by affecting cell cycle exit in the spinal cord. Here, cycling progenitors of early born interneurons were not affected upon loss of DOT1L, whereas this was observed in the cerebral cortex and cerebellum [55, 57]. However, at E12.5 shared genes among different data sets that derived from different DOT1L-depleted parts of the CNS revealed overlapping differentially expressed genes. Two hundred eighty-one altered transcripts after DOT1L deletion were shared between the E14.5 cerebral cortex and E12.5 spinal cord, 183 transcripts were shared between cerebellum P3 and spinal cord E12.5. These shared transcripts fall notably into GO term categories majorly for regulation of mitotic cell cycle for both cortex-spinal cord shared genes and cerebellum-spinal cord shared genes. The former intersect presents enrichment for cell cycle and positive regulation of neuron differentiation, while the latter significantly lists extensively for cell cycle alone. However, in the present study the system appears to be majorly destabilized in the control of migration/localization upon loss of DOT1L. Deletion of DOT1L in the developing cerebellum and cortex also revealed, at least in part, this phenotype. Specifically, *Dot1l*-cKO in the cerebellum [55] affects expression of genes such as *Sema4a*, *Sema5a*, and *Robo1*, while in the cortex [57] disfunction of DOT1L specifically alters the positioning of deep layer neurons (TBR1- or CTIP2-expressing). Integrating our here reported findings on the function of DOT1L for CNS development in a broader context, we propose the view that DOT1L balances progenitor proliferation and their respective programming into specific neuronal subclasses in different parts of the developing CNS including the spinal cord. In addition, DOT1L-mediated transcriptional programs create a surrounding and/or provide capability for neuronal migration, which is also a common feature in all neuroanatomical locations that we studied with regard to DOT1L function.

### Availability of data

The datasets generated and analyzed during the current study are available as RNA-seq raw data in the GEO repository, under the accession number GSE142188.

## Materials and methods extended

### Chick embryos

Fertilized white Leghorn chicken (*Gallus gallus*) eggs were obtained from LSL (Rhein-Main, Germany). For experiments, eggs were incubated in an incubator at approx. 38 °C and 97% humidity, from E0 up to the desired stage.

### qRTPCR

Chick embryos were collected at E2 and staged HH11, HH13+ and HH16 according to the Hamilton-Hamburger stages, the whole neural tubes were dissected in ice-cold PBS and the samples were flash-frozen in liquid nitrogen. Mouse embryos were sacrificed at E9.5-E13.5 and the lumbar spinal cords were dissected in ice-cold DPBS and stored at − 80 °C after flash-freezing in liquid nitrogen. RNA was extracted from the samples with the RNAeasy Mini kit (#74106, Qiagen, Germany), including a DNAse digestion step (#79245, Qiagen, Germany). For chick samples and E9.5-E11.5 murine samples pooling of three samples at the same stage was performed to ensure sufficient material for RNA extraction. cDNA synthesis, qRTPCR (see Table S1 for the primers used) and analysis followed, performed as previously described [60]. GraphPad Prism 6 was used to generate the graphical representation of transcript expression at each timepoint, for mouse embryos p-values were calculated with unpaired, two-tailed Student’s *t*-test.

### Inhibitor treatment and processing of chick embryos

For DOT1L pharmacological inhibition, chicken eggs were incubated until E3 and E3.5. Upon reaching the onset of neurulation (E1), treatment was delivered through a small pinhole in the eggshell, in apical position, at E1 and E2. The treatment was either with DOT1L inhibitor, 5 μm EPZ5676 (S7062, Selleckchem, Munich, Germany) or EtOH as control, dissolved in Locke’s solution (161.31 mM NaCl, 5.968 mM KCl, 2.258 mM CaCl_2_ 2H_2_O). At the desired developmental stage, chick embryos were harvested and fixed overnight in 4% PFA in PBS, washed in PBS and dehydrated in ascending concentrations of EtOH, washed in Roti-Histol (#6640.2, Roth, Karlsruhe, Germany) and embed in paraffin (Leica Biosystems, Nussloch, Germany). Paraffin sectioning was performed at a microtome (Leica) in 10 μm slices mounted on Superfrost slides (Langenbrinck, Emmendingen, Germany), before undergoing deparaffinization and subsequent staining for morphological analysis.

### Hematoxylin-Eosin

Morphological analysis of chick embryos was performed following gradient rehydration of the sections. Hematoxylin-Eosin staining was then performed by placing the slides in a cuvette filled with hematoxylin instant for 30 s. Following 10 min wash under running water and quick washes in H_2_O and 70% EtOH (SigmaAldrich, Taufkirchen, Germany), the slides were then counterstained 1 min in 0.1% Eosin-Y (diluted in 70% EtOH). Upon dehydration with crescent gradient of EtOH, the slides were mounted in Entellan (107,960, Merck Millipore, Darmstadt, Germany).

### Genotyping

Mouse paws were isolated during the dissection of embryos and stored at -20 °C before DNA extraction. DNA was extracted using Quick Extract (Lucigen, WI, USA) according to manufacturer’s instructions. Genotyping of the animals was performed by assessing the presence of the Cre allele and the floxed allele of the *Dot1l* locus, as reported in the Table S1.

### Mouse embryo processing

Mouse embryos were dissected at the desired developmental stage, fixed in 4% PFA (overnight for E18.5 and ISH samples, 20 min for E11.5 and E12.5), washed in PBS and prepared for cryo-embedding in 30% sucrose. The embedding of E18.5 and E13.5 was performed in Tissue-freezing medium (Leica Biosystems, Nussloch, Germany), while E9.5 to E12.5 samples were embedded in 7.5% porcine skin gelatin (G2500-100G, Sigma) and 15% sucrose (4621.1, Roth, Karlsruhe, Germany), and stored at − 80 °C.

Mouse cryosections were cut (Cryostat, Leica Biosystems) at 14 μm for E18.5 and E13.5, at 30 μm for E12.5 to E9.5, at 20 μm for E10.5 and E9.5 for immunolabelling, and stored at − 20 °C before staining.

### Nissl staining

E18.5 mouse cryosections were washed in PBS, then in 0.5% in cresyl violet solution for 5 min. The slides were cleaned with repeated washes in H_2_O supplemented with acetic acid (VWR Chemicals, Bruchsal, Germany), followed by dehydration with ascending EtOH gradient, last washes in Roti-Histol (6640, Roth, Karlsruhe, Germany) and mounting of coverslips with Entellan (107,960, Merck Millipore, Darmstadt, Germany).

### Immunofluorescence

Croysections stored at − 20 °C were treated according to the general protocol for immunostaining that follows, unless specified. The serums used for each antibody are reported in the Table S2, together with the dilutions of primary antibodies. For immunostainings, the cryosections were briefly dried under the chemical hood, washed repeatedly in PBS and permeabilized in 0.1% Triton-X in PBS for 30 min. The sections were then incubated for 60 min at room temperature with blocking solution (specified serum diluted in 0.1% triton/PBS) and incubated overnight at 4 °C with the primary antibodies diluted in the blocking solution. On the second day, washes in 0.1% Triton-X/PBS were performed followed by 2 h incubation with the secondary antibodies diluted in the blocking solution (1:500 Alexa 488/594 donkey anti-rabbit, anti-mouse and anti-goat (Dianova, Hamburg, Germany); Alexa 488 donkey anti-rat and anti-chicken (Jackson Immunoresearch, Suffolk, UK); Alexa 488/568 goat anti-guinea pig (ThermoFisher, MA, USA)). Following secondary antibody incubation, the slides were repeatedly washed in 0.1% Triton-X/PBS and DAPI staining was performed for 5 min. Following further washing in 0.1% Triton-X/PBS and lastly PBS, the slides were mounted with fluorescent mounting medium (#S3023, DAKO, Jena, Germany) and stored in the dark before imaging. Few antibodies required modifications of the described protocol: anti-cleaved CASP3 and anti-GFP were permeabilized with 0.2% Triton-X/PBS, staining anti-ISL1/2 included an antigen retrieval step in citrate buffer with heating step (20 min at 90 °C) between the initial PBS washes and the permeabilization. Staining with anti-ISL1/2 required a different blocking solution (0.3% Triton-X, 0.2% Tween, 10% NGS in PBS) and the antibodies (primary and secondary) were diluted in 10% NGS in 0.1%Triton-X/PBS. Immunostaining for H3K79me2 required an antigen retrieval step in TBS (90 °C for 20 min) and a chromatin-opening treatment with 1 N HCl for 18 min and 2 × 10 minutes washes in Borax (Sodium Borate 0.1 M) prior to permeabilization. Finally, for EdU detection the manufacturer’s instruction manual was used for costaining with OLIG3 (#C10337, Click-iT™ EdU Cell Proliferation Kit for Imaging, PA, USA).

### Imaging, quantification and figures preparation

Immunofluorescence images were generated using an Axioplan M2 fluorescent microscope (Zeiss, Germany), paired with an Apotome.2 module. Hemi-sections of spinal cord were analyzed for quantification and distributional analysis. In either case, at least 4 hemi-sections from the lumbar spinal cord were taken per animal (specified per staining), for at least 3 biological replicates (animals of each condition). The counts per hemi-sections were averaged for each animal and the means of the biological replicates between control and *Dot1l-*cKO condition were statistically tested with unpaired Student’s t-test using GraphPad Prism 6. The images were processed for optimal visualization and quantification by enhancing colors on the ZENblue software, aligned in the dorsoventral axis on Inkscape and quantified (for number and distribution) on ImageJ. The absolute cell numbers were used to calculate the means. Inkscape was also used to assemble the paper figures. A schematic representation of the counting process for distribution is reported in Figure S6.

## Supplementary information

**Additional file 1: Table S1.** List of used probes and primers.

**Additional file 2: Table S2.** List of Antibodies.

**Additional file 3: Figure S1.** DOT1L expression in mouse and chicken spinal cord; DOT1L inhibition associates with NTD in chicken. (**A**) ISH for *Dot1l* transcripts in lumbar spinal cord hemi-sections from E9.5 to E13.5 of control or wildtype embryos. Cord or hemicord profiles highlighted by dotted black line. Scale bars: 100 μm. (**B**) qRTPCR analysis of *DOT1L* in untreated chick spinal cords at different embryonic stages (HH11-HH16, comparable to mouse E9.0, E9.5 and E10.0) normalized to HH11 (n = 1). (**C**) Representative bright-field whole mount pictures of control and *Dot1l*-cKO littermates from E9.5 to E11.5, including magnification from a side that corresponds to white squares in the whole embryo images. (**D**) Hematoxylin-eosin staining on lumbar neural tube paraffin-embedded sections of chick embryos on E3 from the controls and after DOT1L inhibition. Right panels: magnifications of insets boxed on the left. Scale bar: 100 μm. (**E**) Relative occurrence of observed phenotypes in control and inhibitor-treated samples at E3 and E3.5 (control E3 n = 11, DOT1L-inhibited E3 n = 15, control E3.5 n = 20, DOT1L-inhibited E3.5 n = 22). Percentages of observed phenotypes are represented.

**Additional file 4: Figure S2.** GFP reporter assay displaying CRE activity, *Dot1l*-cKO embryonic lethality and unaltered progenitor domains. (**A**) Immunostaining of GFP reporter for CRE activity in lumbar spinal cord at E12.5. (**B**) Occurrence of observed genotypes (control: +/+;*Dot1l*^*fl/+*^ or +/+;*Dot1l*^*fl/fl*^, heterozygous cKO: Cre/+;*Dot1l*^*fl/+*^, mutant: Cre/+;*Dot1l*^*fl/fl*^) at different embryonic stages (E12.5, E13.5, E18.5 and P0). E12.5 n = 1 (10 embryos), E13.5 n = 4 (35 embryos), E18.5 n = 4 (23 embryos), P0 n = 5 (46 embryos). (**C**) Representative immunostainings of SOX2 (red) in E11.5 lumbar spinal cord sections of control and cKO littermates. DAPI (gray) in counterstaining. (**D**) Representative immunostaining of Nestin (NES, red) counterstained by DAPI (grey) in E9.5 control and *Dot1l*-cKO spinal cords. (**E**) Representative immunostaining of EdU (green) and OLIG3 (red) in E9.5 control and *Dot1l*-cKO spinal cords. (**F-G**) Immunostainings of NES and EdU with OLIG3 in E10.5 control and *Dot1l*-cKO E10.5 littermates. Hemicord profiles highlighted by dotted white line. Scale bar: 100 μm.

**Additional file 5: Figure S3.** Increased cell death is restricted to E18.5 in *Dot1l*-cKO. (**A, C, F, G**) Representative immunostainings for cleaved CASP3 (red) in lumbar areas of control and of mutant littermates at prenatal stage (E18.5 in **A**) and over neurogenesis (E11.5 in **C**, E12.5 in **F** and E13.5 in **G**) counterstained by DAPI (gray). Hemicord profiles highlighted by dotted white line. Scale bars: 100 μm. (**B, E, I**) Quantitative analyses of immunostainings for CASP3 and DAPI-dense pyknotic nuclei at E18.5, E11.5 and E13.5. Quantification represented with mean ± SEM. P-value was calculated with unpaired, two-tailed Student’s *t*-test. ** *p* < 0.01. At E18.5, different hemi-sections were counted for each n (*n* = 4), for a total of 14 hemi-sections for the control condition and 16 hemi-sections for the mutant condition. At E11.5, 4 hemi-sections per n were counted (*n* = 3), while at E13.5 5 hemi-sections per n were counted (*n* = 3). (**D, H**) Density plots for quantified cell death (CASP3 and pyknotic nuclei) respectively at E11.5 and E13.5.

**Additional file 6: Figure S4.** DOT1L does not affect dI3 migration at E11.5. (**A**) Density plots for dI3 interneurons at E11.5, representing TLX3-single stained cells as shown in Fig. 7a. Density plot projections analyzed by multivariate analysis for Hotelling’s two-sample square test; † p = 0.08. Stars are reported on the Y axis (dorsoventral, DV) or X axis (mediolateral, ML) according to values on the individual axes. (**B**) Quantitative analysis of immunostainings for dI3 TLX3-single stained cells at E11.5. Quantifications represented with mean ± SEM. P-values were calculated with unpaired, two-tailed Student’s *t*-test. 4 hemi-sections were counted for each n (*n* = 3).

**Additional file 7: Figure S5.***Dot1l*-cKO transcriptome reveals a shift towards interneuron differentiation at the expense of proliferation. (**A, left panel**) Heatmap for differentially expressed genes in mutant littermates intersected with a published gene list for identifiers specific for differentiated interneuron populations [68]⁠. Color-coding based on TPM z-score, scale to the top left side. (**A, right panel**) Annotation of differentiated domain-specificity relative to the genes intersected in the heatmap, based on published domain specific genes [68]. Highlighted in yellow, markers for dI1 (*Lhx2*), dI3 (*Isl1*) and dI5 (*Lmx1b*) with differential expression previously analyzed in the study. (**B, C, D, E**) Heatmaps representing DEG in *Dot1l*-cKO intersected with different GO terms, respectively axon guidance, neuronal migration, positive regulation of cell cycle and regulation of synapse structure/activity. (**F**) GO enrichment analysis of genes shared by DEG of *Dot1l*-cKO in P3 cerebellum to the left [55] and E14.5 cortex to the right [57] with E12.5 lumbar spinal cord. Adjusted p-value and scales of gene ratio reported to the top left side. Threshold for enrichment analysis: adjusted p < 0.1.

**Additional file 8: Figure S6.** Schematic representation of quantification of neuronal subtype distribution. (A) In both control and *Dot1l*-cKO samples, matching hemicords were selected. Lowest point of the section in the center was defined as origin (black circle). For normalization among sections, the maximal width from the central canal to the most extreme external border (blue bar), and the maximal height from the lowest point of the spinal cord to the rooftop was measured (red bar). For each specified cell population in study, distance (green bar) and angle (darker green area) of the singular cell bodies (violet stars) were measured relative from the origin. (B) For the statistical analysis, each counted cell body was projected on the x-axis (blue stars) and y-axis (yellow stars). (C) At minimum 4 hemisections from three biological replicates were assessed in terms of cell body counts and their respective projection on the x- and y-axis, results from the different sections cumulated and superimposed (black arrow) to plot the cell distributions and to test for statistically significant shifts along the axes. (D) Stacking of all measured cell bodies as indicated in (C) was cumulated and represented in a color-coded distributional map (red highest cell density, blue lowest cell density). Color-coded densities refer to one experiment assessing distribution of one specific subpopulation. The relative values for red and blue vary between individual cell populations and should not be compared for different markers.

## Abbreviations

cKO: Conditional knock-out
CNS: Central nervous system
DEG: Differentially expressed genes
dI(X): Dorsal interneuron
DOT1L: Disruptor Of Telomeric Silencing 1-Like protein
dp(X): Dorsal progenitor
E(X): Embryonic day – days after mating (mice) or beginning of incubation (chicken)
GO: Gene Ontology
H3K79me: Histone H3 Lysine 79 methylation
Hcy: Homocysteine
MN: Motor neuron
NTD: Neural tube defect
p(X): Ventral progenitor
P(X): Postnatal days (mouse age)
pMN: Motor neuron progenitor
qRTPCR: Quantitative Reverse Transcription Polymerase Chain Reaction
RNA-seq: RNA sequencing
SEM: Standard error of mean
TPM: Transcripts per kilobase million
V(X): Ventral interneuron
