# Supplementary material for: Differentiation and localization of interneurons in the developing spinal cord depends on DOT1L expression
Source: Mol Brain. 2020 May 29;13:85. doi: 10.1186/s13041-020-00623-3 (PMC7260853; doi:10.1186/s13041-020-00623-3)
Supplement: Supplementary file 1 — Additional file 1: Table S1. List of used probes and primers. [file 13041_2020_623_MOESM1_ESM.docx]

**Table S1: List of used probes and primers**

| **Gene** | **Species** | **Use** | **Forward** | **Reverse** |
| --- | --- | --- | --- | --- |
| Gad65 | Mouse | ISH | Provided by Wahle lab (Bochum) | |
| Lhx5 | Mouse | ISH | CGAGAATGAGGAACAGAACTCC | GAGGGCTTCTAAGATGTGCTTG |
| Reln | Mouse | ISH | Described in (57) | |
| Dot1l | Mouse | ISH | Described in (57) | |
| Dot1l | Mouse | Genot. | GCCTACAGCCTTCATCATTC | CCCATACAGTACTCACCGGAT |
| Wnt1cre | Mouse | Genot. | ACAGCGAACCATGCTGCCTG | CATGTCCATCAGGTTCTTGC |
| Dot1l | Chick | qPCR | ACTGAAGGAGCGATTTGCCA | TCCACGACACAGAGCCTTTC |
| Gapdh | Chick | qPCR | TGACCACTGTCCATGCCATC | CTTTCCCCACAGCCTTAGCA |
| Dot1l | Mouse | qPCR | TGAGGCTCAAGTCGCCTGT | GCATCATGGTGCTTGTCGTA |
| Gapdh | Mouse | qPCR | CGGCCGCATCTTCTTGTG | TGACCAGGCGCCCAATAC |
