# Supplementary material for: Differentiation and localization of interneurons in the developing spinal cord depends on DOT1L expression
Source: Mol Brain. 2020 May 29;13:85. doi: 10.1186/s13041-020-00623-3 (PMC7260853; doi:10.1186/s13041-020-00623-3)
Supplement: Supplementary file 2 — Additional file 2: Table S2. List of Antibodies. [file 13041_2020_623_MOESM2_ESM.docx]

**Table S2: List of Antibodies**

| **Antibody** | **Species** | **Company** | **Purchase number** | **Dilution** | **Blocking** |
| --- | --- | --- | --- | --- | --- |
| anti-H3K79me2 | Rabbit | Abcam, Cambridge, UK | ab3594 | 1/500 | 10% NDS |
| anti-KI67 | Rabbit | Abcam, Cambridge, UK | ab15580 | 1/200 | 5% HS |
| anti-BRN3A | Mouse | Santa Cruz Biotechnology | sc-8429 | 1/200 | 5% HS |
| anti-OLIG3 | Guinea Pig | T. Müller/Birchmeier |  | 1/10’000 | 5% HS |
| anti-LHX2 | Rabbit | Abcam, Cambridge, UK | ab184337 | 1/200 | 5% HS |
| anti-LHX9 | Rabbit | Abcam, Cambridge, UK | ab224357 | 1/100 | 5% HS |
| anti-EVX1 | Mouse | DSHB, IA, USA | 99.1-3A2 | 1/200 | 5% HS |
| anti-FOXD3 | Guinea Pig | T. Müller/Birchmeier | #3 | 1/10’000 | 5% HS |
| anti-TLX3 | Guinea Pig | T. Müller/Birchmeier | #6 | 1/20’000 | 5% HS |
| anti-LMX1B | Rabbit | T. Müller/Birchmeier | #20 | 1/10’000 | 5% HS |
| anti-ISL1/2 | Mouse | DSHB, IA, USA | 39.4D5 | 1/200 | 10% NGS |
| anti-LBX1 | Guinea Pig | T. Müller/Birchmeier | #706 | 1/20’000 | 5% HS |
| anti-PAX2 | Rabbit | Thermofisher | 71-6000 | 1/250 | 5% HS |
| anti-FOXP1 | Goat | R&D | AF4534 | 1/500 | 5% HS |
| anti-GFP | Chicken | Abcam, Cambridge, UK | ab13970 | 1/500 | 10% NDS |
| anti-SOX2 | Rabbit | Abcam, Cambridge, UK | ab97959 | 1/200 | 10% NDS |
| anti-NES | Mouse | Abcam, Cambridge, UK | ab6142 | 1/1000 | 2% NGS, 2.5% BSA |
| anti-cleaved-CASP3 | Rabbit | Cell Signaling, MA, USA | 9661S | 1/500 | 10% NDS |
