# Supplementary figures and images for: Differentiation and localization of interneurons in the developing spinal cord depends on DOT1L expression
Source: Mol Brain. 2020 May 29;13:85. doi: 10.1186/s13041-020-00623-3 (PMC7260853; doi:10.1186/s13041-020-00623-3)

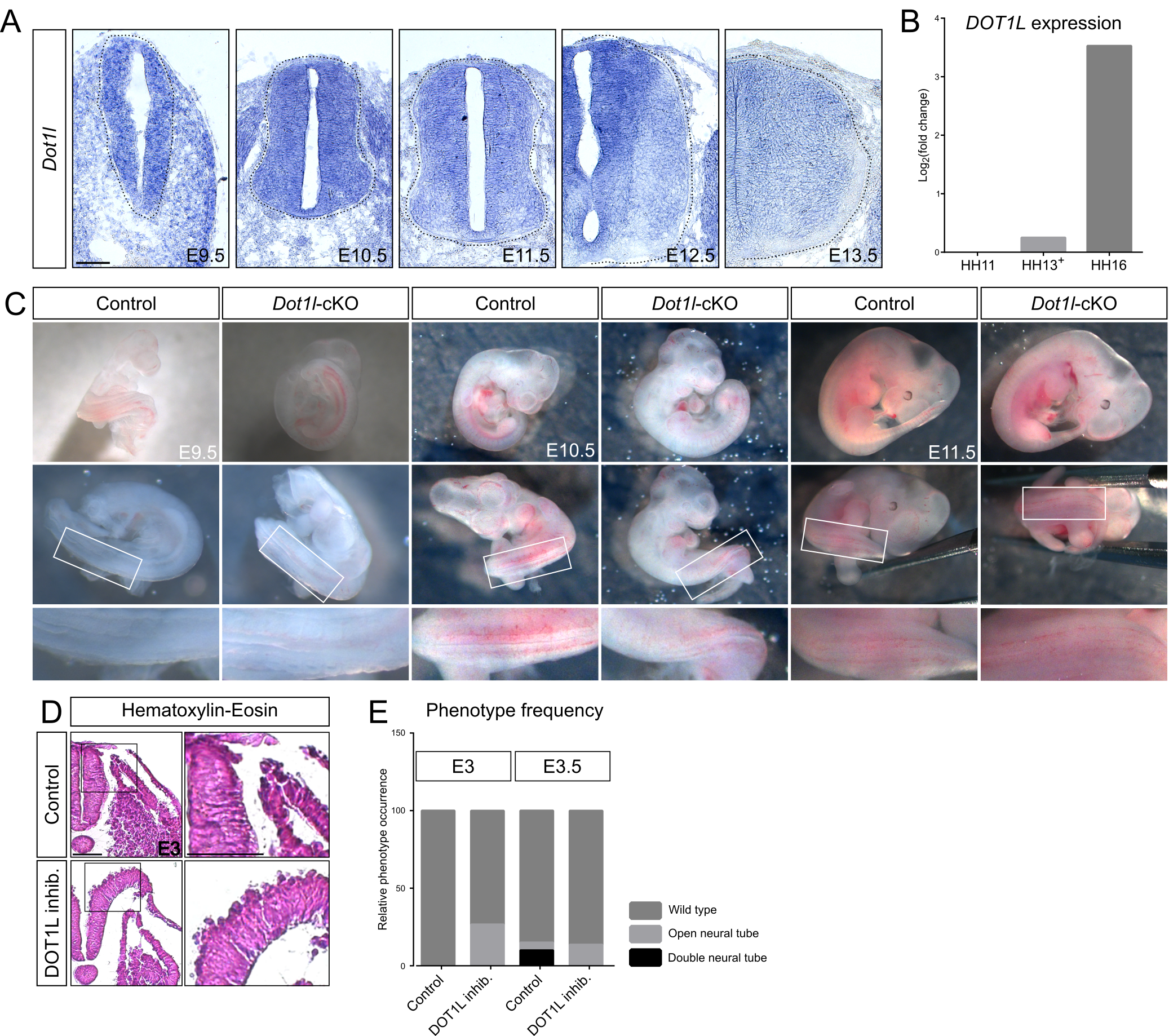

Supplement: Supplementary file 3 — Additional file 3: Figure S1. DOT1L expression in mouse and chicken spinal cord; DOT1L inhibition associates with NTD in chicken. (A) ISH for Dot1l transcripts in lumbar spinal cord hemi-sections from E9.5 to E13.5 of control or wildtype embryos. Cord or hemicord profiles highlighted by dotted black line. Scale bars: 100 μm. (B) qRTPCR analysis of DOT1L in untreated chick spinal cords at different embryonic stages (HH11-HH16, comparable to mouse E9.0, E9.5 and E10.0) normalized to HH11 (n = 1). (C) Representative bright-field whole mount pictures of control and Dot1l-cKO littermates from E9.5 to E11.5, including magnification from a side that corresponds to white squares in the whole embryo images. (D) Hematoxylin-eosin staining on lumbar neural tube paraffin-embedded sections of chick embryos on E3 from the controls and after DOT1L inhibition. Right panels: magnifications of insets boxed on the left. Scale bar: 100 μm. (E) Relative occurrence of observed phenotypes in control and inhibitor-treated samples at E3 and E3.5 (control E3 n = 11, DOT1L-inhibited E3 n = 15, control E3.5 n = 20, DOT1L-inhibited E3.5 n = 22). Percentages of observed phenotypes are represented. [file 13041_2020_623_MOESM3_ESM.png]

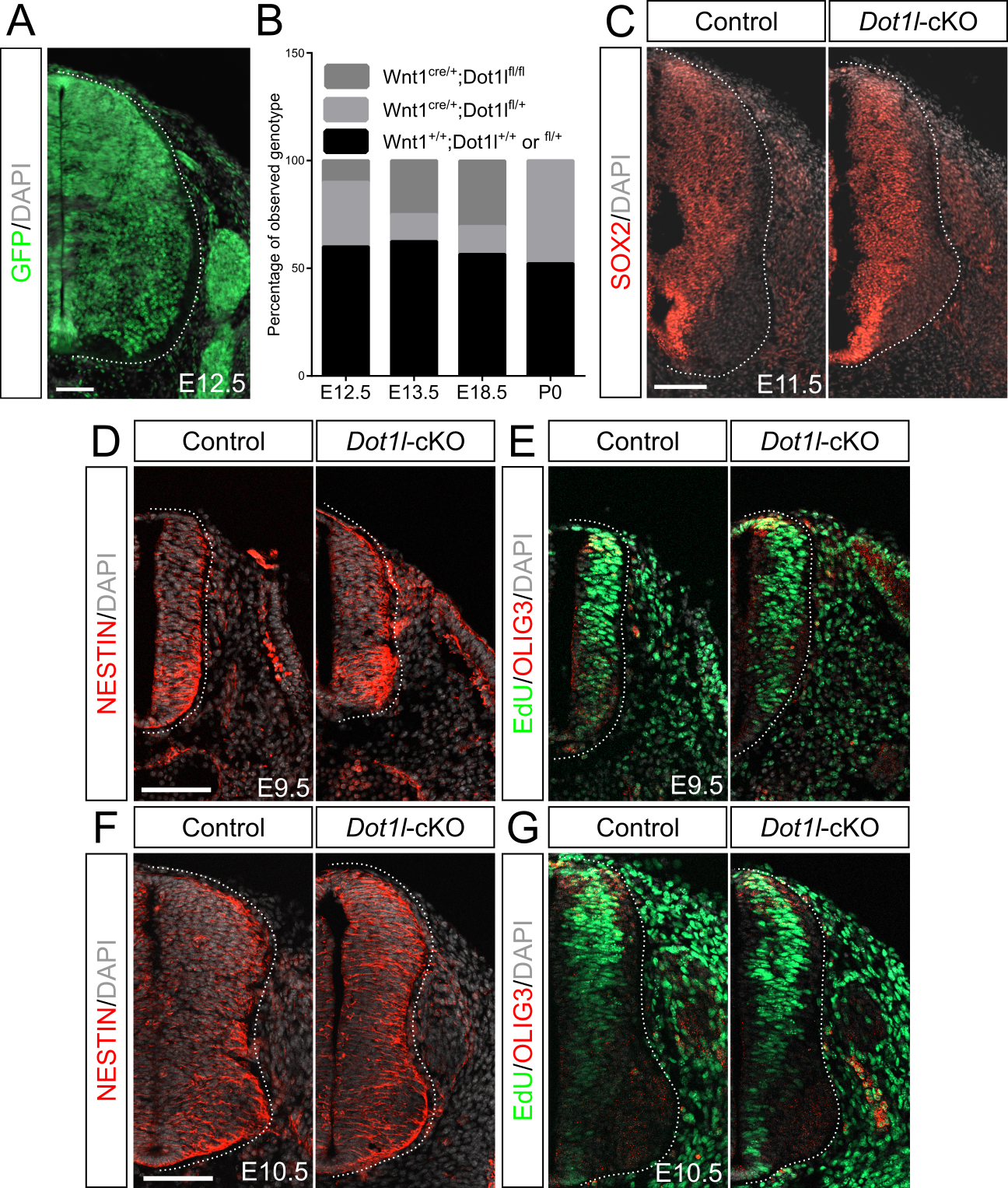

Supplement: Supplementary file 4 — Additional file 4: Figure S2. GFP reporter assay displaying CRE activity, Dot1l-cKO embryonic lethality and unaltered progenitor domains. (A) Immunostaining of GFP reporter for CRE activity in lumbar spinal cord at E12.5. (B) Occurrence of observed genotypes (control: +/+;Dot1lfl/+ or +/+;Dot1lfl/fl, heterozygous cKO: Cre/+;Dot1lfl/+, mutant: Cre/+;Dot1lfl/fl) at different embryonic stages (E12.5, E13.5, E18.5 and P0). E12.5 n = 1 (10 embryos), E13.5 n = 4 (35 embryos), E18.5 n = 4 (23 embryos), P0 n = 5 (46 embryos). (C) Representative immunostainings of SOX2 (red) in E11.5 lumbar spinal cord sections of control and cKO littermates. DAPI (gray) in counterstaining. (D) Representative immunostaining of Nestin (NES, red) counterstained by DAPI (grey) in E9.5 control and Dot1l-cKO spinal cords. (E) Representative immunostaining of EdU (green) and OLIG3 (red) in E9.5 control and Dot1l-cKO spinal cords. (F-G) Immunostainings of NES and EdU with OLIG3 in E10.5 control and Dot1l-cKO E10.5 littermates. Hemicord profiles highlighted by dotted white line. Scale bar: 100 μm. [file 13041_2020_623_MOESM4_ESM.png]

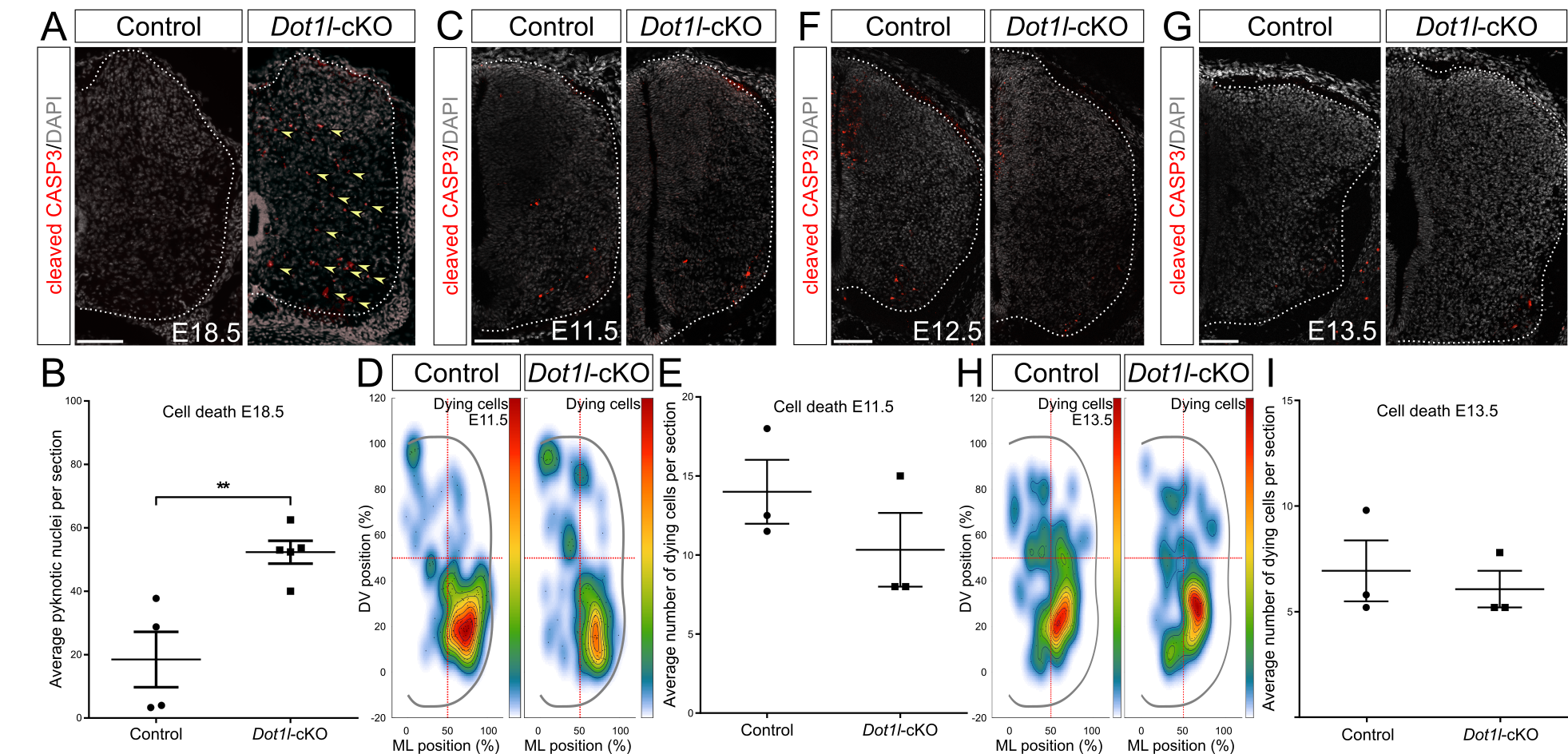

Supplement: Supplementary file 5 — Additional file 5: Figure S3. Increased cell death is restricted to E18.5 in Dot1l-cKO. (A, C, F, G) Representative immunostainings for cleaved CASP3 (red) in lumbar areas of control and of mutant littermates at prenatal stage (E18.5 in A) and over neurogenesis (E11.5 in C, E12.5 in F and E13.5 in G) counterstained by DAPI (gray). Hemicord profiles highlighted by dotted white line. Scale bars: 100 μm. (B, E, I) Quantitative analyses of immunostainings for CASP3 and DAPI-dense pyknotic nuclei at E18.5, E11.5 and E13.5. Quantification represented with mean ± SEM. P-value was calculated with unpaired, two-tailed Student’s t-test. ** p < 0.01. At E18.5, different hemi-sections were counted for each n (n = 4), for a total of 14 hemi-sections for the control condition and 16 hemi-sections for the mutant condition. At E11.5, 4 hemi-sections per n were counted (n = 3), while at E13.5 5 hemi-sections per n were counted (n = 3). (D, H) Density plots for quantified cell death (CASP3 and pyknotic nuclei) respectively at E11.5 and E13.5. [file 13041_2020_623_MOESM5_ESM.png]

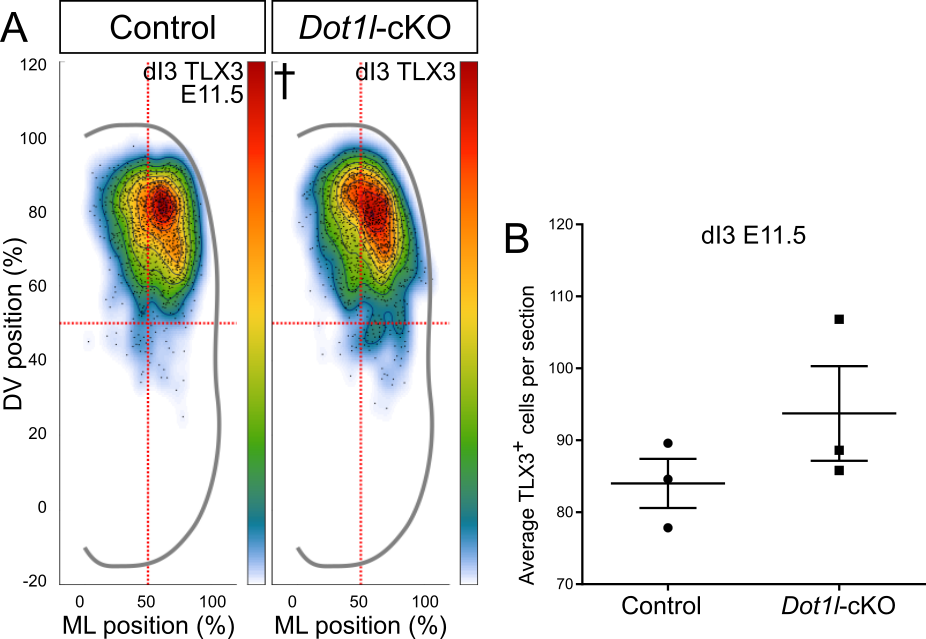

Supplement: Supplementary file 6 — Additional file 6: Figure S4. DOT1L does not affect dI3 migration at E11.5. (A) Density plots for dI3 interneurons at E11.5, representing TLX3-single stained cells as shown in Fig. 7a. Density plot projections analyzed by multivariate analysis for Hotelling’s two-sample square test; † p = 0.08. Stars are reported on the Y axis (dorsoventral, DV) or X axis (mediolateral, ML) according to values on the individual axes. (B) Quantitative analysis of immunostainings for dI3 TLX3-single stained cells at E11.5. Quantifications represented with mean ± SEM. P-values were calculated with unpaired, two-tailed Student’s t-test. 4 hemi-sections were counted for each n (n = 3). [file 13041_2020_623_MOESM6_ESM.png]

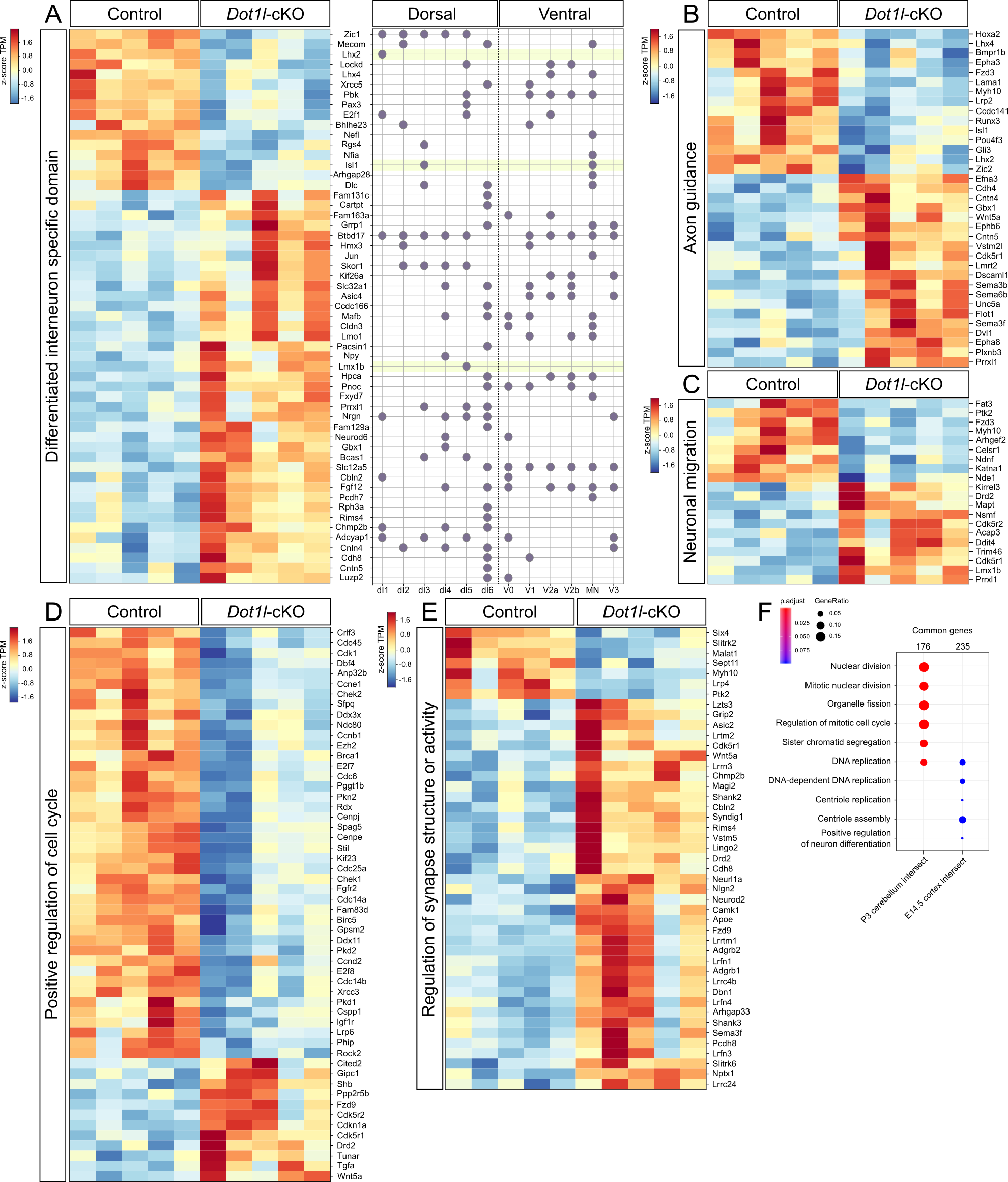

Supplement: Supplementary file 7 — Additional file 7: Figure S5.Dot1l-cKO transcriptome reveals a shift towards interneuron differentiation at the expense of proliferation. (A, left panel) Heatmap for differentially expressed genes in mutant littermates intersected with a published gene list for identifiers specific for differentiated interneuron populations [68]⁠. Color-coding based on TPM z-score, scale to the top left side. (A, right panel) Annotation of differentiated domain-specificity relative to the genes intersected in the heatmap, based on published domain specific genes [68]. Highlighted in yellow, markers for dI1 (Lhx2), dI3 (Isl1) and dI5 (Lmx1b) with differential expression previously analyzed in the study. (B, C, D, E) Heatmaps representing DEG in Dot1l-cKO intersected with different GO terms, respectively axon guidance, neuronal migration, positive regulation of cell cycle and regulation of synapse structure/activity. (F) GO enrichment analysis of genes shared by DEG of Dot1l-cKO in P3 cerebellum to the left [55] and E14.5 cortex to the right [57] with E12.5 lumbar spinal cord. Adjusted p-value and scales of gene ratio reported to the top left side. Threshold for enrichment analysis: adjusted p < 0.1. [file 13041_2020_623_MOESM7_ESM.png]

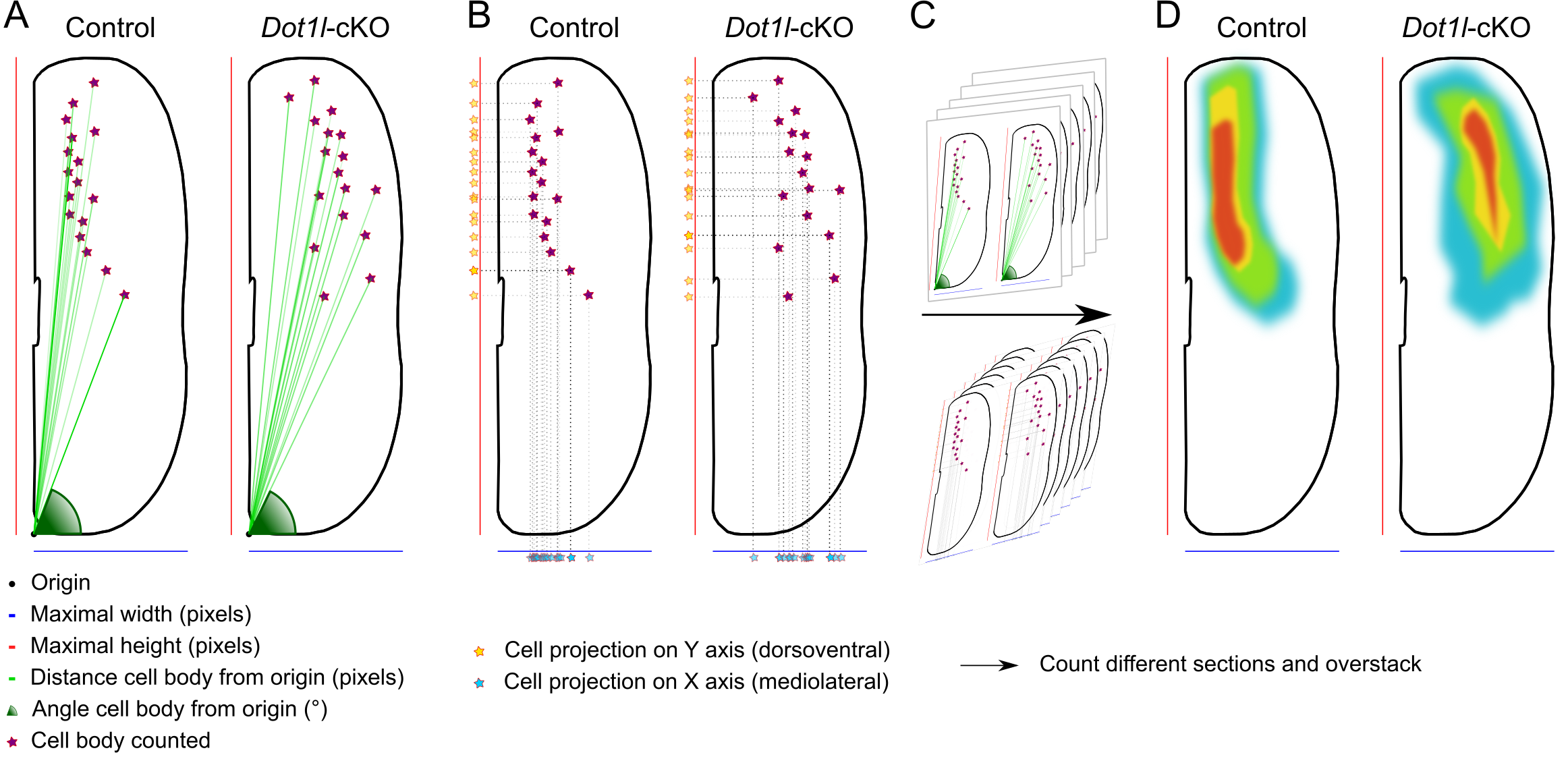

Supplement: Supplementary file 8 — Additional file 8: Figure S6. Schematic representation of quantification of neuronal subtype distribution. (A) In both control and Dot1l-cKO samples, matching hemicords were selected. Lowest point of the section in the center was defined as origin (black circle). For normalization among sections, the maximal width from the central canal to the most extreme external border (blue bar), and the maximal height from the lowest point of the spinal cord to the rooftop was measured (red bar). For each specified cell population in study, distance (green bar) and angle (darker green area) of the singular cell bodies (violet stars) were measured relative from the origin. (B) For the statistical analysis, each counted cell body was projected on the x-axis (blue stars) and y-axis (yellow stars). (C) At minimum 4 hemisections from three biological replicates were assessed in terms of cell body counts and their respective projection on the x- and y-axis, results from the different sections cumulated and superimposed (black arrow) to plot the cell distributions and to test for statistically significant shifts along the axes. (D) Stacking of all measured cell bodies as indicated in (C) was cumulated and represented in a color-coded distributional map (red highest cell density, blue lowest cell density). Color-coded densities refer to one experiment assessing distribution of one specific subpopulation. The relative values for red and blue vary between individual cell populations and should not be compared for different markers. [file 13041_2020_623_MOESM8_ESM.png]
